# Resource‐driven colonization by cod in a high Arctic food web

**DOI:** 10.1002/ece3.7025

**Published:** 2020-11-23

**Authors:** Edda Johannesen, Nigel G. Yoccoz, Torkild Tveraa, Nancy L. Shackell, Kari E. Ellingsen, Andrey V. Dolgov, Kenneth T. Frank

**Affiliations:** ^1^ Institute of Marine Research Nordnes Norway; ^2^ Department of Arctic and Marine Biology UiT The Arctic University of Norway Tromsø Norway; ^3^ Norwegian Institute for Nature Research (NINA) Fram Centre Langnes Norway; ^4^ Ocean Sciences Division Bedford Institute of Oceanography Darthmouth Canada; ^5^ Polar Branch of the Federal Russian Research Institute of Fisheries and Oceanography (PINRO) Murmansk Russia; ^6^ Murmansk State Technical University branch of Federal State Educational Institution of Higher Education Murmansk Russia; ^7^ Tomsk State University Tomsk Russia

**Keywords:** abiotic, Barents Sea, biotic, hierarchical design, marine food webs, range expansion, spatial distribution, stomach data

## Abstract

1. Climate change is commonly associated with many species redistributions and the influence of other factors may be marginalized, especially in the rapidly warming Arctic.
2. The Barents Sea, a high latitude large marine ecosystem in the Northeast Atlantic has experienced above‐average temperatures since the mid‐2000s with divergent bottom temperature trends at subregional scales.
3. Concurrently, the Barents Sea stock of Atlantic cod *Gadus morhua,* one of the most important commercial fish stocks in the world, increased following a large reduction in fishing pressure and expanded north of 80°N.
4. We examined the influence of food availability and temperature on cod expansion using a comprehensive data set on cod stomach fullness stratified by subregions characterized by divergent temperature trends. We then tested whether food availability, as indexed by cod stomach fullness, played a role in cod expansion in subregions that were warming, cooling, or showed no trend.
5. The greatest increase in cod occupancy occurred in three northern subregions with contrasting temperature trends. Cod apparently benefited from initial high food availability in these regions that previously had few large‐bodied fish predators.
6. The stomach fullness in the northern subregions declined rapidly after a few years of high cod abundance, suggesting that the arrival of cod caused a top‐down effect on the prey base. Prolonged cod residency in the northern Barents Sea is, therefore, not a certainty.

## Graphical abstract

We examined the influence of food availability and temperature on cod expansion using a comprehensive data set on cod stomach fullness stratified by subregions characterized by divergent temperature trends. The greatest increase in cod occupancy occurred in three northern subregions with contrasting temperature trends. Here, cod apparently benefited from initial high food availability with few large‐bodied fish predators prior to the arrival of cod.

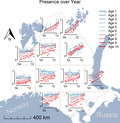

## 1 INTRODUCTION

Poleward species range expansions are commonly interpreted as direct consequences of climate warming (Parmesan & Yohe, 2003). Since temperature directly influences physiological processes in ectotherms, a direct simple link between climate change and range shifts is often assumed (Comte & Olden, 2017; Kingsolver et al., 2011). However, a large number of studies on a wide variety of organisms have demonstrated the importance of biotic interactions in shaping distributions reviewed in Ref. (Louthan et al., 2015; Svenning et al., 2014; Wisz et al., 2013) involving predation (Trekels & Vanschoenwinkel, 2019), competition (Aguilera et al., 2019), and parasitism (Bozick & Real, 2015). Fewer empirical studies have demonstrated how food resources impact distribution shifts (but see, e.g., Davies et al., 2019; Stewart et al., 2014).

Here, we take advantage of a period of extensive survey coverage to examine the range expansion of the highly migratory Northeast Arctic (NEA) stock of cod (*Gadus morhua*). The principal feeding grounds of NEA cod are the Barents Sea, a large (~1.6 million km^2^), high latitude shelf sea (~70° to 80°N) bordering the polar basin. The Arctic Ocean region has been warming more rapidly than other ocean regions (Screen & Simmonds, 2010) and has become more favorable for the growth and survival of sub‐Arctic species (Alabia et al., 2018) such as cod. In the Barents Sea, a warming trend, resulting in decreased sea ice cover, began in the 1970s (ICES, 2019). From around 2005 and onwards, water temperatures at several depths of the Barents Sea have been significantly warmer compared with the period from 1970 to 2000 (ICES, 2019; Lind et al., 2018). Concurrently, the distribution of cod expanded (Fall et al., 2018; Ingvaldsen et al., 2015), reaching 80°N and thus appeared to reflect the general warming trend in the region (Fossheim et al., 2015).

Cod is a dominant generalist predator in the Barents Sea food web (Bogstad et al., 2000; Holt et al., 2019). Mobile predators may shift distributions rapidly in response to environmental cues such as local depletion of food resources (Ims & Yoccoz, 1997). Food shortage may thus motivate the search for new areas where rich resources compensate for the energy spent during relocation (Yackulic et al., 2017). From 2004 to 2013, cod spawning stock biomass increased nearly fourfold reaching a historic high level (ICES, 2018; Kjesbu et al., 2014). Increased food competition as the stock increased to record high levels may have led to the expansion of cod into new areas in search of abundant prey.

Large‐bodied fish species in marine ecosystems have experienced declines in abundance and distribution as well as changes in age/size structure over the last decades and centuries, often as a consequence of unsustainable management and poor fishing practices (Worm & Tittensor, 2011). The fishing pressure on NEA cod was reduced by 40% in the mid‐2000s (Figure S1) and was followed by an increase in both abundance and spatial distribution. Knowledge of range expansion and contraction is essential in the management of harvested fish stocks (MacCall, 1990; Marshall & Frank, 1994; Petitgas, 1998; Shackell et al., 2005), and for a given system, it is useful to assess the relative role of climate, resource management, and food web interactions in driving the spatiotemporal dynamics (Pörtner & Peck, 2010). A general pattern across systems is that species abundance is positively related to range size (Gaston & Blackburn, 2000) which is often taken as evidence for density‐dependent habitat selection (Fretwell & Lucas, 1970). However, density dependence is not a mechanism per se (Krebs, 1995, 2002), but can be explained by underlying factors that are frequently more difficult to assess, especially at the large spatial scale of commercially exploited fish stocks.

Determination of the mechanisms underlying the recent northward expansion of NEA cod was made possible by the implementation of a broad‐scale ecosystem survey of the entire Barents Sea shelf during the summer feeding period from 2004 to 2013 when the system was largely ice‐free (ICES, 2019). During the main feeding season, cod consume a large array of smaller‐bodied species, the most important prey species being capelin *Mallotus villosus* (Johannesen et al., 2012). Other diet items include bottom‐dwelling fish (e.g., juvenile cod and haddock *Melanogrammus aeglefinus*), pelagic fish (e.g., Polar cod *Boreogadus saida*, herring *Clupea harengus*), benthic invertebrates (e.g., shrimp *Pandalus borealis*), and zooplankton (e.g., krill, *Euphausiacae*). A comprehensive data set on cod stomach fullness obtained during the ecosystem surveys was used as a proxy for prey availability. The stomach fullness integrates the total amount of prey that an individual has recently encountered and successfully captured and is therefore a suitable index of per capita prey availability.

**Figure 1:**
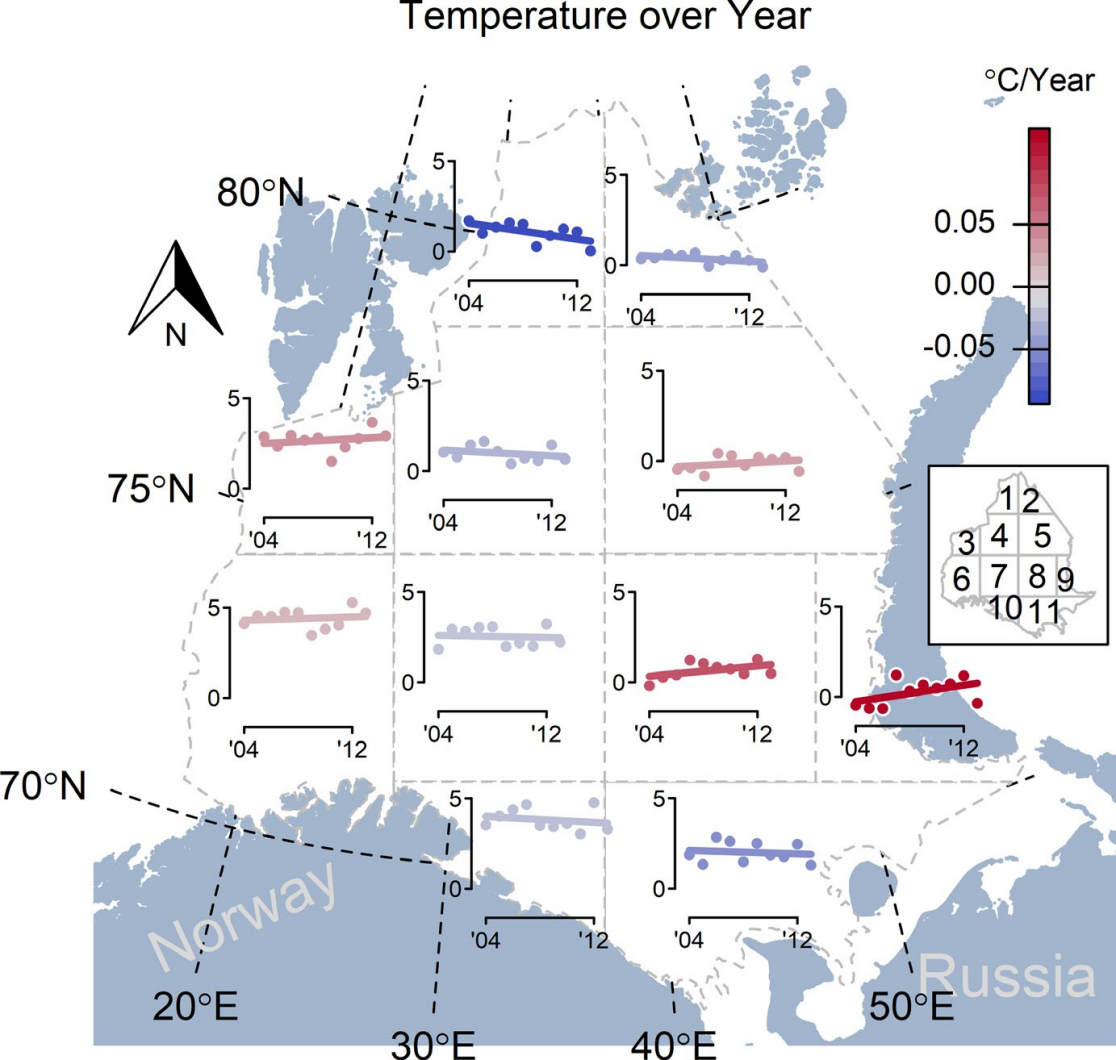
Annual, subregion bottom temperatures averaged across sets where CTDs (conductivity, temperature, depth sensor) were deployed. The color is proportional to the yearly change (blue—cooling, red—warming)

Despite the general warming trend documented in the Arctic Ocean region, the Barents Sea exhibits pronounced temporal and spatial variation in bottom temperature (Ellingsen et al., 2020; ICES, 2019). Divergent temporal trends in ocean bottom temperature were evident in eleven subregions within the Barents Sea (Ellingsen et al., 2020) facilitating the analysis of cod expansion in a quasi‐experimental hierarchical design (Shadish et al., 2002) (Figure 1). Expansion of cod aged 1–10 years was analyzed in relation to stomach fullness within the hierarchical design permitting the evaluation of the relative importance of food and temperature as drivers of the recent cod expansion.

## 2 MATERIALS AND METHODS

### 2.1 Sampling procedure and data

Cod were sampled during the joint Russian Norwegian annual ecosystem survey in the Barents Sea initiated by the Institute of Marine Research (IMR), Norway, and the Knipovich Polar Research Institute of Marine Fisheries and Oceanography (PINRO) of the Russian Federation. The survey was initiated in 2004 and has been conducted annually in August–September (duration 39–57 days)—the time of the year when, on average, the Barents Sea has minimal ice cover. We assessed data from 2004 to 2013—years when cod increased in abundance and a period of extensive survey coverage (Figures S1, S2).

Trawl stations were set out in a fixed regular grid design with stations ~60 km apart with some minor exceptions (Figure S2). All vessels used a Campelen 1,800 bottom trawl (see, e.g., Bergstad et al. (2018) for details on equipment and bottom trawl configuration). The number of trawl stations per year ranged from 310 (in 2010) to 580 (in 2005) and covered an area of ~1.3 million km^2^ (68–82°N, 14–57°E); a total of 3,416 trawl sets were evaluated. Bottom temperature measurements were obtained from the deployment of a CTD (conductivity, temperature, and depth) probe adjacent to each trawl set in the grid.

All cod from each trawl set were measured for length (cm), and one cod from each 5 cm length group was later aged based on otolith readings. The oldest cod was 17 years; however, due to limited numbers, cod aged 10 years and older were combined and subsequently referred to as age 10 cod. Stomach contents for one cod from each 5 cm length group from each trawl set were weighed to the nearest milligram. Cod stomach fullness (CSF) was used as an index of prey availability and was standardized for the different length groups by dividing stomach content weight by the length of the cod cubed and multiplied by 10^4^ (Figure S3; Dalpadado & Bogstad, 2004; Lilly & Fleming, 1981). Cod occupancy (CO) was measured as the proportion of trawl sets with cod present. Cod occupancy and cod abundance were highly correlated (Figure S4).

### 2.2 Study design

We used only the continental shelf of the Barents Sea because the area west and north of Svalbard is topographically very different—it is a shelf break with large heterogeneity in depth and temperature (see Bergstad et al., 2018). Also, sampling in this area has been very variable. We used eleven subregions (Figure 1) previously defined by Ellingsen et al. (2020) who assessed the relative effect of temperature and cod on changes in spatial beta diversity of the demersal fish community over time. The subregions were defined by interpolating the spatial variation for large cod and temperature on a joint grid for each year using an additive model with a bivariate tensor product smooth of the spatial coordinates, and a smooth term for depth (Wood, 2006). For temperature, a smooth effect of sampling date was included as temperature increased in the beginning of each survey period. Interpolated values of cod and temperature (standardized to 200 m depth) at each point in the grid were then regressed against year to describe the spatial variability in trends. Finally, 11 subregions of approximately similar sizes were defined guided by maps of the regression coefficients. The approach is further detailed in Ellingsen et al. (2020). We also binned the Barents Sea into subregions using 4 × 4 and 5 × 5 geographical grid designs in order to assess the robustness of our approach (Table S5).

### 2.3 Statistical analyses

Generalized linear mixed models were used to analyze how cod occupancy (CO), measured as the proportion of trawl sets with cod present, and cod stomach fullness (CSF) in each of the eleven subregions changed over time. Year was used as a fixed, linear effect, to represent the average temporal change, transformed to year 2004 so that the estimated intercept represented the stomach fullness and proportion of trawls with cod at the beginning of the study. The need for transformation of CSF was assessed using the Box–Cox transformation family using the MASS library, the parameters being estimated using profile log‐likelihood (Venables & Ripley, 1999). We therefore transformed CSF as “log (CSF + 0.02),” hereafter referred to as CSF (Figure S3). Prior to analyses, bottom temperature data were standardized to 200 m depth and a common median survey date (3 September) for each year.

Random intercepts and slopes for the year effect were used to analyze the variation among age classes (10 levels) and subregions (11 levels). Additive effects of age and subregions were considered, as well as the interaction age:subregion in order to model different responses among age classes in the various subregions. The global (most complex) model for the response variables CO and CSF *Y_ijk_* (i: age, j: subregion, k: year) was therefore:

*Y_ijk_* is distributed with mean $\mu_{\mathrm{ijk}}$, and the mean $\mu_{\mathrm{ijk}}$, conditionally on the random effects *a_ij_* and *b_ij_*, is given by

$$f\left(\mu_{\mathrm{ijk}}|{a_{\mathrm{ij}},b}_{\mathrm{ij}}\right)={\alpha +\beta {\mathrm{Year}}_{k}+a}_{\mathrm{ij}}+b_{\mathrm{ij}}{\mathrm{Year}}_{k},$$

The function f is the logit for CO, and identity for CSF, α is the model intercept and β is the slope associated with the year effect, and a_ij_ and b_ij_ are normally distributed with a mean of 0 and variances $\sigma_{a}^{2}$ and $\sigma_{b}^{2}$, respectively, i (i = 1, …, 10) and j (j = 1, …, 11), indexing age classes and subregions, respectively. Simpler models assumed that the random effects a and b depended on additive effects of age and subregions, age (*a_i_*, *b_i_*) or subregions (*a_j_*, *b_j_*) only, or were removed (i.e., no variation among age‐groups or subregions in slopes or intercepts). Models with varying slopes but constant intercepts were not evaluated.

For CO, both generalized linear mixed models (GLMMs) with a binomial distribution (using the number of trawls with cod present or absent as the response) and a linear mixed model on the proportions were considered. Both approaches gave similar results, and we present estimates from the model using GLMMs. Models were checked using the residuals plotted by subregion and age. Model performance was assessed using WAIC and leave‐one‐out cross‐validation (LOO) (Vehtari et al., 2017). Models were run using the “*rstanarm*” library which implements generalized linear mixed models in a Bayesian framework using *Stan* (Gabry & Goodrich, 2018; Gelman & Hill, 2007). By default, all *rstanarm* modeling functions run four randomly initialized Markov chains, each for 2,000 iterations (including a burn‐in period of 1,000 iterations that is discarded). *Stan* estimates an effective sample size for each parameter, which plays the same role in the Markov chain Monte Carlo central limit theorem (MCMC CLT) as the number of independent draws plays in the standard central limit theorem (CLT). Convergence statistics, R‐hat (Brooks & Gelman, 1997; Gelman & Rubin, 1992), were less than 1.005, and effective sample size was greater than 781 for all focal parameters. We used *Stan's* default weakly informative priors with mean of zero and unscaled standard deviation of 10 for intercept and 2.5 for coefficients—adjusted for, for example, stomach fullness to 8.62 and 0.75, respectively, for the best model (Gabry & Goodrich, 2018). See Table S1 for complete information regarding priors.

A Bayesian approach was also used to quantify the association between the degree of cod stomach fullness at the start of the survey (2004), the change in ocean temperature, and rate of change in cod occupancy for the different age classes and subregions. We used models with age‐specific and sub‐region‐specific coefficients for the year effect for all response variables. We estimated the correlation for each age class and across subregions between the estimated stomach fullness in 2004 (i.e., the intercept as year was defined as 0 for 2004) and the rate of change in CSF and CO (proportion of stations with cod present). That is (with i indexing age classes, j subregions, and k years):

$$E\left[\log\left({\text{CSF}}_{\mathrm{ijk}}+0.02\right)\right]=\alpha_{\text{CSF},\;ij}+\beta_{\text{CSF},\;ij}{\text{Year}}_{k}$$

$$\log\text{it[}p_{\text{CO}\;ijk}]=\alpha_{\text{CO},\;ij}+\beta_{\text{CO},\;ij}{\text{Year}}_{k}$$

and we estimated Cor(α_CSF_ *_,ij_*, β_CO,_ *_ij_*) for each age class i and across subregions within the MCMC based on the chains for each parameter α_CSF,_ *_ij_* and β_CO,_ *_ij_*. This approach assumed independence between the error terms for CSF and CO.

The same model structure was used to calculate the correlation between estimated change in ocean temperature and change in proportion of stations with cod present for each age class. That is:

$$E\left[{\text{Temp}}_{\mathrm{ijk}}\right]=\alpha_{\text{Temp},\;ij}+\beta_{\text{Temp},\;ij}{\text{Year}}_{k}$$

$$\log\text{it[}p_{\mathrm{COijk}}]=\alpha_{CO,\;ij}+\beta_{CO,\;ij}{\text{Year}}_{k}$$

and we estimated Cor(β_Temp,_ *_ij_*, β_CO,_ *_ij_*) for each age class i and across subregions. By implementing a Bayesian approach, integration of the uncertainty associated with the estimation of intercepts and slopes was achieved. The model was implemented in *rjags* (Plummer, 2016), a R interface for jags (Plummer, 2003). Parameter estimates and credibility intervals were based on three chains of 20,000 MCMC permutations, with the first 10,000 permutations in each chain discarded as burn‐in. To avoid autocorrelation among the permutations, only every fifth permutation was used, resulting in a total of 6,000 sampled permutations in each analysis. A weakly informative normal prior distribution was used for regression intercepts and slopes, with a mean of 0 and standard variation of 10 for all coefficients. Convergence statistics, R‐hat, was less than 1.009, and effective sample size was greater than 235 for all focal parameters.

## 3 RESULTS

The coldest water temperatures in the Barents Sea were observed in the northern and northeastern subregions. Warming was most pronounced in the eastern and central Barents Sea, while other subregions had stable temperature conditions or cooling trends during the 2004 to 2013 interval (Figure 1).

**Figure 2:**
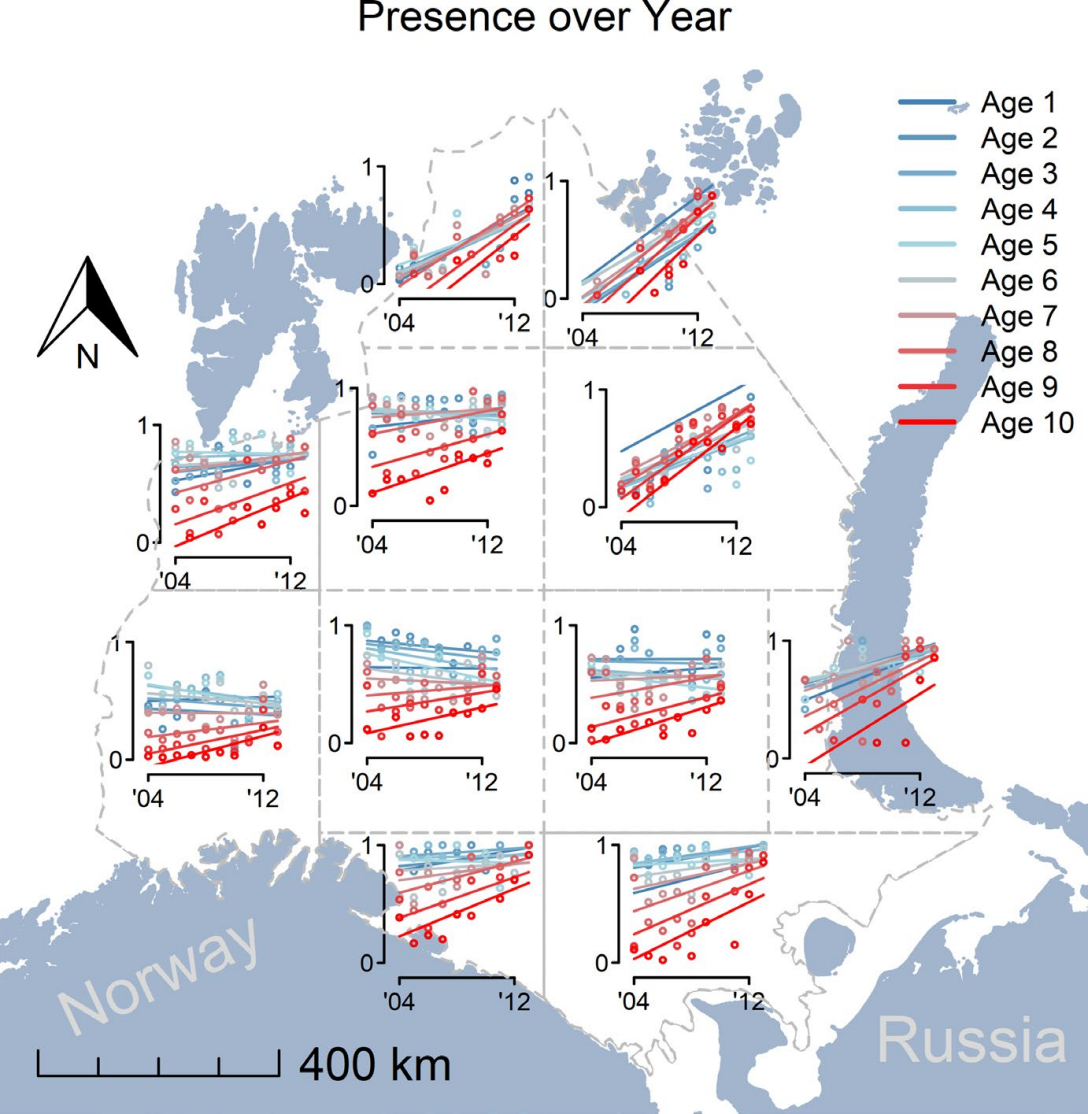
Change in occupancy of cod (annual proportion of sets with cod relative to all trawl sets in the subregion) over time. The lines are the estimated slopes from the most parsimonious model (Table S1), and the dots represent the observed occupancy (ranges from 0 to 1)

Cod colonization and rapidly increasing occupancy were observed in the north and northeast subregions (subregions 1, 2, and 5; Table S2) of the Barents Sea (Figures 2, S2). The cod response was not restricted to any single age‐group, rather all ages (ages 1–10) increased. Some differences among age classes did occur, for example, highest expansion of 9‐year‐old cod, followed 10‐, 8‐, 1‐, and 7‐year‐old cod (Figure 2). The northern subregions were nearly devoid of cod during the first five survey years in contrast to the central and southern subregions where increasing occupancy over time was evident but at much slower rates and seen primarily among the older age‐groups (Figure 2).

**Figure 3:**
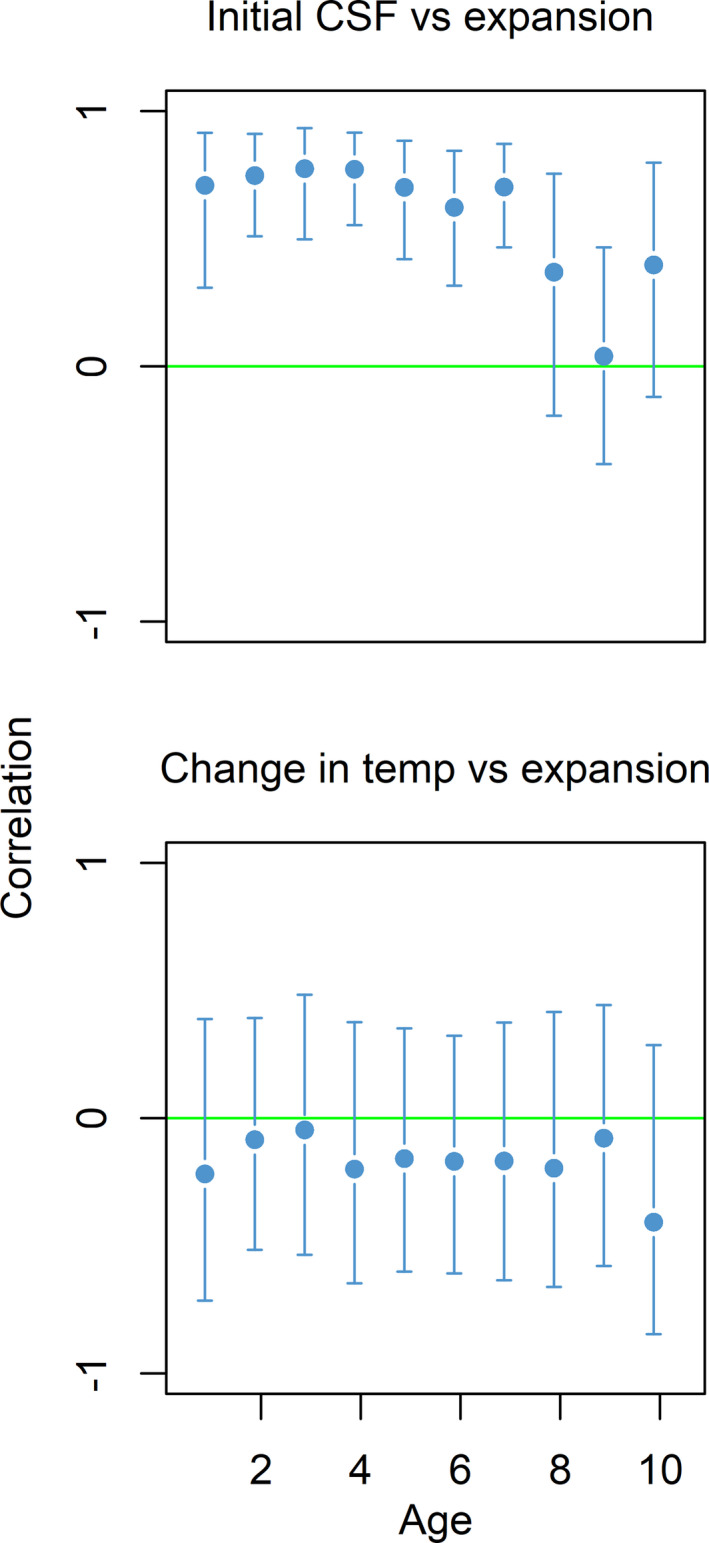
Upper: estimated correlation (mean ± 95% credible interval) for each age class between subregional change in occupancy and stomach fullness (CSF) in 2004. Lower: estimated correlation (mean ± 95% credible interval) for each age class between region specific change in occupancy and change in temperature

There was no evidence for an effect of temperature change on occupancy, given that occupancy increased in subregions with contrasting temperature trends (Figures 1, 2). Changes in bottom temperature (Figure 1) were unrelated to the observed expansion of cod for all age‐groups (Figure 3).

**Figure 4:**
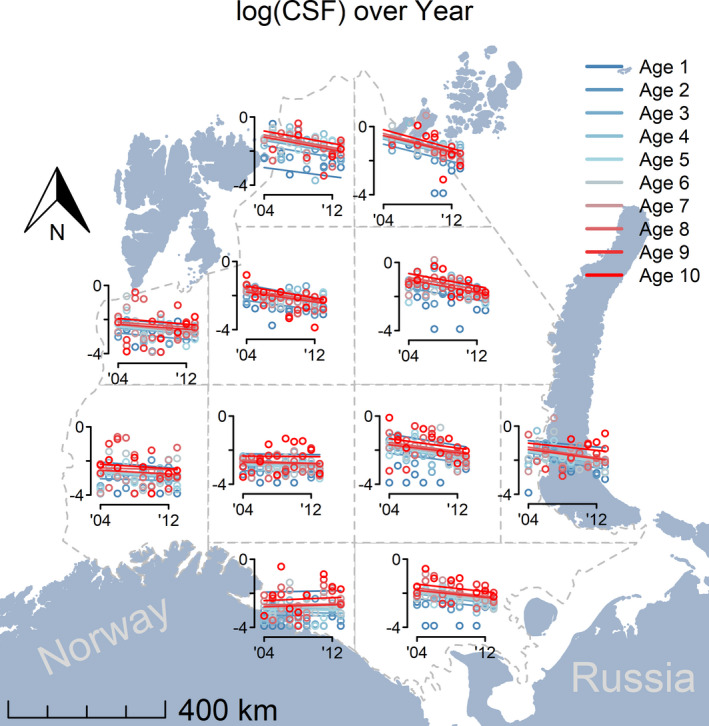
Change in cod stomach fullness (CSF) over time by subregion. The lines are the estimated slopes from the most parsimonious model (Table S1), and the dots represent the observed stomach fullness by age and year

The feeding success based on cod stomach fullness (CSF) was initially high and subsequently decreased in the northern subregions (subregions 1, 2, and 5; Table S3). Stomach fullness remained constant and was generally low in the southwest (Figure 4). In subregions with higher initial occupancy of cod, the initial stomach fullness was relatively low.

The mechanism underlying cod expansion appears to be the ambient feeding conditions given the significant relationship between the rate of cod expansion and initial cod stomach fullness (Figure 3). The positive relationship between sub‐region‐specific initial stomach fullness and the change in occupancy by subregion was evident for all ages (Figure 3). The same results were evident from the alternative geographical grid designs (Table S5).

Statistical models, ignoring differences in occupancy and stomach fullness changes among subregions, performed more poorly in comparison with those models taking such variation into account (Table S4). The most parsimonious models also included age class differences in expansion and changes in stomach fullness, and interaction between age and subregion (Table S4).

## 4 DISCUSSION

The recent expansion of cod in the Barents Sea represents the northernmost occurrence of this species. We have shown that cod expanded fastest into subregions wherever food was abundant, regardless of whether the temperature was decreasing, stable, or increasing. A simple, direct link between temperature increase and range expansion of marine fishes is often assumed (Comte & Olden, 2017). No evidence of such a simple relationship between temperature and cod occurrence was found. The relative importance of active (migration) over passive (drift and local mortality) processes in shaping cod distribution increases with cod age and size (Ciannelli et al., 2020), Nevertheless, positive relationships between sub‐region‐specific initial stomach fullness and the change in occupancy by subregion was evident for all ages.

The subregional changes in temperature were relatively small, compared with temperature differences between the warmer southwestern and the colder northeastern Barents Sea (ICES, 2019), and compared with the temperature tolerance of cod (Righton et al., 2010). Cod have been captured in temperatures ranging from −1.5 to 19°C, growing fastest at temperatures 8–10°C (Righton et al., 2010), which is warmer than the current temperatures in the Barents Sea (ICES, 2019). Temperature tolerance is, however, much narrower for cod in spawning condition (Righton et al., 2010). Mature NEA cod are therefore restricted to the warmer waters along the coast of Northern Norway during the spawning season in spring—separated in time and space by many months and 1,000 kms from the main expansion area in the far north. Our results suggest that the cold, northern Barents Sea is used for feeding only when the stock levels are high. Comparable survey data from the main feeding season for earlier periods are lacking.

Earlier studies based on data from January–March (the Norwegian Russian winter survey) covering the south‐central Barents Sea have found temperature effects on cod distributions (Ottersen et al., 1998). The timing of the winter survey coincides with the spawning migration of cod. Accordingly, in a recent study utilizing winter survey data, Fall et al. (2018) found that the effect of temperature on distribution differed between immature and mature cod. However, an unknown proportion of immature cod appear to be distributed outside the area surveyed in winter (Johansen et al., 2013). Our results are based on one season and do not fully entail the spatial dynamics of Barents Sea cod. Nevertheless, our study covers the main feeding season of cod. At this time of year, food appeared to be the main driver of cod expansion of cod of all ages as cod peaked in abundance.

Our study and others have demonstrated that NEA cod are highly opportunistic and can rapidly respond to environmental changes through alternate migration patterns (Dolgov, 2016; Harden Jones, 1968; Johannesen et al., 2012). The initial occurrence of cod in the most northern subregions was associated with the highest stomach fullness indices relative to all other subregions. We assume that high stomach fullness in cod is caused by high encounter rates between individual cod and prey, suggesting that expanding cod could quickly exploit a previously underutilized prey base. During the expansion period of cod, a similar northwards expansion of its main prey capelin (*Mallotus villosus*) occurred, associated with an increase in population size from a collapsed state in 2004–2007 to substantially higher levels in 2008–2013 (Fall et al., 2018). The cod expansion may, however, only partly be driven by capelin, since capelin are restricted to the northwestern Barents Sea which correspond to our subregions 1, 3, and 4 (Fall et al., 2018), whereas cod mainly increased in subregions 1, 2, and 5 (Figure 1).

Low abundances of large predatory fishes other than cod characterized the northern Barents Sea prior to the recent arrival of cod (Frainer et al., 2017; Kortsch et al., 2015). Marine mammals and seabirds are present in the area, but whereas the body condition of the most abundant marine mammals declined during the cod expansion, cod growth remained relatively stable during the same period despite a record large population (Bogstad et al., 2015; ICES, 2018). Compared with other top predators in the Barents Sea, cod use a wider range of prey, and habitats—a likely contributor to its high productivity during the study period.

Cod is an important capture fish, ranking 9th of marine capture fishes globally (FAO, 2018). The Barents Sea is a productive area for cod; presently about two thirds of the global cod catches are from the Barents Sea. In other areas, fishing activity leading to depletion and distributional changes has been observed for cod (Carson et al., 2017; Shackell et al., 2005). The recent large‐scale increase in NEA cod abundance resulted from a combination of reduced mortality of older fish due to reduced fishing pressure (Figure S1), combined with high productivity of the population (Kjesbu et al., 2014). The high productivity of the population has been attributed to climatic effects and the overall warm conditions in the recent years (Kjesbu et al., 2014). In addition, our results illustrate how the initially high prey resources in the northernmost subregions of the Barents Sea allowed cod to successfully increase in abundance while expanding and profiting from favorable conditions there.

Cod have a strong top‐down structuring effect on the biological communities of shelf ecosystems in the North Atlantic, most dramatically demonstrated by the regime shift associated with the collapse of cod populations in the Northwest Atlantic (Ellingsen et al., 2015; Frank, 2005; Shackell et al., 2012; Shackell & Frank, 2007). Based on cod stomach fullness, the food availability in the main expansion area and in the Barents Sea as a whole decreased over the course of our study likely due to intensive cod predation (Table S3). Notable is the fact that small demersal Arctic fishes, Arctic hyperiids, and the pelagic Arctic species polar cod have declined during the study years (CAFF, 2017; Frainer et al., 2017; Johannesen et al., 2017; Stige et al., 2019). Thus, the feeding advantage that initially existed in the northern waters declined and may herald an end to the expansion. If the prey base is not sufficiently replenished, the increase in cod in the northern Barents Sea during the study period may represent a transient effect, similar to what has been observed for the Baltic cod (Casini et al., 2016; Eero et al., 2012, 2015).

Although the Barents Sea is a relative simple and species‐poor system, strong seasonality in productivity and species distributions as well as indirect interactions makes it difficult to evaluate the overall impact of climate variability (Stige et al., 2019) and cod on the prey base (Howell & Filin, 2013). For instance, cod feed substantially on the main prey (krill and hyperiids) of its prey (polar cod and capelin) is cannibalistic and is both prey of and in competition with the dominant marine mammals in the area (Bogstad et al., 2000). Future research aimed at the recent declining phase in cod should provide more insight into the long‐term effect of cod predation in the Northern Barents Sea. The years following the present study, the size of the cod stock has declined (Figure S1). In the years 2014–2019, the survey coverage was poorer, but in 2019 cod appear to have retracted from the main expansion area (Figure S5). Despite increased primary productivity associated with decreased ice cover in the northern Barents Sea (Dalpadado et al., 2014), it will likely take several years for slow‐growing Arctic prey species to recover from their recent bout of high cod predation pressure (Frainer et al., 2017). If Arctic species are not replaced by boreal species suitable as cod prey, the beneficial feeding conditions for cod in northern Barents Sea will not be quickly restored, and therefore, prolonged cod residency in the northern Barents Sea is not a certainty.

## CONFLICT OF INTEREST

The authors declare no conflict of interest.

## AUTHOR CONTRIBUTIONS

**Edda Johannesen:** Conceptualization (equal); Data curation (equal); Writing‐original draft (lead); Writing‐review & editing (lead). **Nigel G. Yoccoz:** Conceptualization (equal); Formal analysis (lead); Methodology (lead); Writing‐original draft (supporting); Writing‐review & editing (supporting). **Torkild Tveraa:** Conceptualization (equal); Formal analysis (supporting); Visualization (lead); Writing‐original draft (supporting); Writing‐review & editing (supporting). **Nancy L. Shackell:** Conceptualization (equal); Writing‐original draft (equal); Writing‐review & editing (equal). **Kari E. Ellingsen:** Conceptualization (equal); Funding acquisition (lead); Project administration (lead). **Andrey V. Dolgov:** Conceptualization (equal); Data curation (equal); Writing‐original draft (supporting); Writing‐review & editing (supporting). **Kenneth T. Frank:** Conceptualization (equal); Writing‐original draft (equal); Writing‐review & editing (equal).

## Supporting information

Supplementary Material

## Data Availability

Data by subregions were archived in Dryad. The data access DOI number is https://doi.org/10.5061/dryad.3tx95x6dx.
