# Supplementary material for: Resource‐driven colonization by cod in a high Arctic food web
Source: Ecol Evol. 2020 Nov 23;10(24):14272–81. doi: 10.1002/ece3.7025 (PMC7771159; doi:10.1002/ece3.7025)
Supplement: Supplementary file 1 — Supplementary Material [file ECE3-10-14272-s001.pdf]

Supporting Information

Resource-driven colonization by cod in a high Arctic food web

Running title: Expansion by cod in the Arctic

Edda Johannesen[1], Nigel G. Yoccoz[2,3], Torkild Tveraa[3], Nancy L. Shackell[4], Kari E. Ellingsen[3], Andrey V. Dolgov[5,6,7], Kenneth T. Frank[3,4]

1 Institute of Marine Research, P. O. Box 1879, 5817 Nordnes, Norway

2 Department of Arctic and Marine Biology, UiT The Arctic University of Norway, 9037 Tromsø, Norway

3 Norwegian Institute for Nature Research (NINA), Fram Centre, P.O. Box 6606 Langnes, 9296 Tromsø, Norway

4 Ocean Sciences Division, Bedford Institute of Oceanography, P.O. Box 1006, Darthmouth, Nova Scotia B2Y 4A2, Canada

5 Polar Branch of the Federal Russian Research Institute of Fisheries and Oceanography (PINRO), Academik Knipovich Street 6, Murmansk, Russia, 183038

6 Murmansk State Technical University branch of Federal State Educational Institution of Higher Education, Sportivnaya Street 13, Murmansk, Russia, 183010

7 Tomsk State University, 36 Lenin Avenue, Tomsk, Russia, 634050

Table S1. Overview of weakly informative prior distributions used to analyse temporal changes in cod occupancy (CO) and cod stomach fullness (CSF). We used the default parameterization of rstanarm, which rescales prior distributions. All random effects as well as the residual variation are parameterized by the standard deviations sigmas and have the same exponential prior distribution.

Cod Occupancy

	Distribution	Mean	SD	Adjusted SD
Intercept	Normal	0	10	
Coefficient (year)	Normal	0	2.5	0.87

Sigmas: we used the default prior in rstan.glmer, defined by ~ decov(reg. = 1, conc. = 1, shape = 1, scale = 1)

Cod Stomach Fullness

	Distribution	Mean	SD	Adjusted SD
Intercept	Normal	0	10	8.621
Coefficient (year)	Normal	0	2.5	0.753
Sigmas	Exponential	1(rate)		0.862

Table S2. Yearly change in logit of cod occupancy for each sub-region. 95% credible intervals not overlapping 0 are shown in bold

Sub-region	Median	2.5.% CI	97.5% CI
1	**0.30**	0.22	0.39
2	**0.50**	0.40	0.60
3	0.07	-0.02	0.16
4	0.08	-0.005	0.17
5	**0.24**	0.15	0.33
6	-0.01	-0.09	0.08
7	0.01	-0.07	0.10
8	**0.11**	0.03	0.20
9	0.01	-0.07	0.10
10	**0.15**	0.06	0.23
11	**0.22**	0.13	0.32

Table S3. Yearly change in Cod Stomach Fullness for each sub-region. Significant changes are marked in bold.

Sub-region	Mean	2.5.% CI	97.5% CI
1	**-0.085**	-0.117	-0.048
2	**-0.126**	-0.173	-0.079
3	**-0.051**	-0.084	-0.020
4	**-0.059**	-0.090	-0.026
5	**-0.105**	-0.145	-0.067
6	-0.009	-0.044	0.027
7	**-0.038**	-0.071	-0.006
8	**-0.076**	-0.113	-0.045
9	-0.017	-0.052	0.019
10	-0.028	-0.061	0.004
11	**-0.068**	-0.100	-0.032

Table S4. Comparison of alternative models explaining Cod Occupancy and Stomach Fullness, based on the design-based regions and two Information Criteria, Leave-One Out (LOOIC) and Widely Applicable (WAIC). All models included a fixed (average) temporal trend, but differed in how intercepts (defined in the models as Year=2004) and slopes (=temporal trends) varied among Age groups and sub-regions, either additively or interactively (Age:Region).

**Response variable**	**Intercept**			**Variable slope**				
**Occupancy**	Age	Region	Age:Region	Age	Region	Age:Region	LOOIC	WAIC
	x	X	x	x	x	X	6170	6134
	x	X	x	x			7084	7069
	x	X		X	x		7172	7172
	x	X	X	X	x		7173	7173
	x	X	x	x			7425	7414
	x	X	x				8129	8128
	x	X			x		8576	8574
	x	X		x			10442	10443
	x	X					10444	10443
		X					10446	10446
	x						11235	11235
**Stomach fullness**	x	X	x	x	x		1802.7	1802.1
	x	X	x	x	x	x	1804.3	1802.7
	x	X		x	x		1806.1	1806.0
	x	X	x		x		1808.3	1807.8
	x	X			x		1811.0	1810.9
	x	X	x	x			1824.5	1824.0
	x	X		x			1827.1	1827.0
	x	X	x				1830.0	1829.6
	x	X					1831.4	1831.4
		X					2030.8	2030.8
	x						2382.2	2382.2

Table S5: Estimated correlations between intercept for CSF analyses (estimated 2004 CSF) and increase in CO, according to three different designs: 1) based on changes on cod and temperature, 2) on a 4x4 regular grid, and 3) on a 5x5 regular grid. Estimates with 95% credible intervals in parenthesis.

	Cod-Temp Design	*4x4 grid*	*5x5 grid*
Age 1	*0.67 [-0.05; 0.91]*	*0.61 [-0.05; 0.90]*	*0.13 [-0.36; 0.61]*
Age 2	*0.63 [0.34, 0.84]*	*0.62 [0.34; 0.83]*	*0.62 [0.35; 0.82]*
Age 3	*0.78 [0.53; 0.92]*	*0.77 [0.54, 0.92]*	*0.66 [0.44; 0.83]*
Age 4	*0.69 [0.37; 0.87]*	*0.65 [0.37; 0.86]*	*0.47 [0.19; 0.71]*
Age 5	*0.44 [0.06; 0.73]*	*0.51 [0.10; 0.79]*	*0.34 [-0.05; 0.68]*
Age 6	*0.74 [0.50, 0.89]*	*0.73 [0.50; 0.89]*	*0.60 [0.34; 0.81]*
Age 7	*0.66 [0.45; 0.83]*	*0.66 [0.46; 0.83]*	*0.64 [0.41; 0.82]*
Age 8	*0.56 [0.28; 0.77]*	*0.55 [0.29; 0.78]*	*0.22 [-0.12; 0.55]*
Age 9	*0.24 [-0.25; 0.63]*	*0.22 [-0.24; 0.62]*	*0.36 [-0.03; 0.66]*
Age 10	*0.64 [0.22; 0.85]*	*0.63 [0.27; 0.86]*	*0.09 [-0.79; 0.88]*

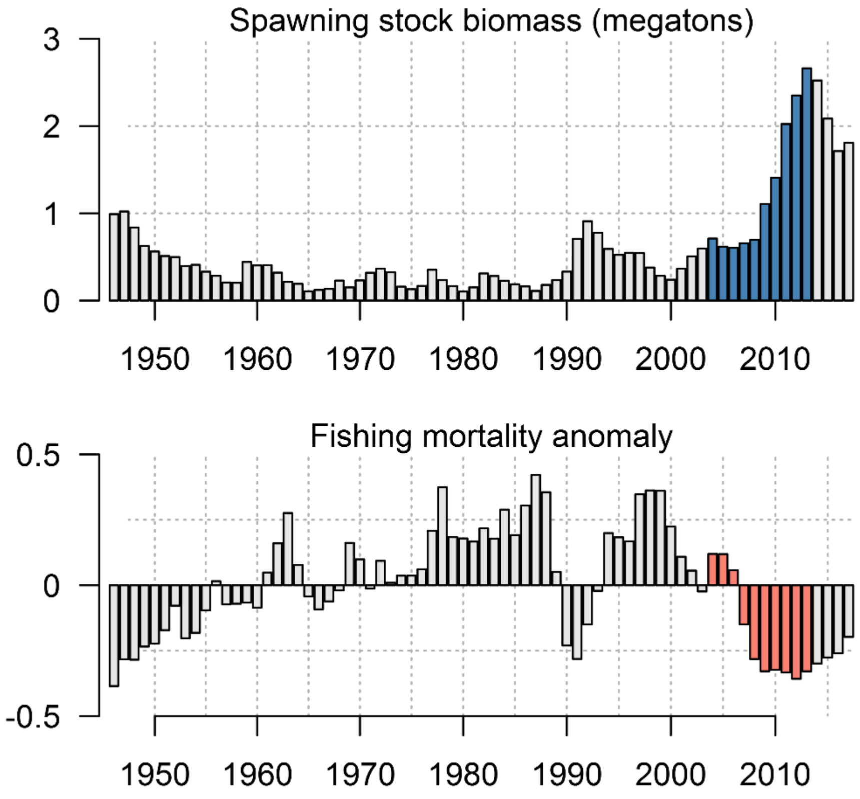

Figure S1 Cod spawning stock biomass and Fishing mortality anomaly (F – F average) is indicated in blue and red, respectively.

Data from: ICES (2018) Report of the Arctic Fisheries Working Group (AFWG). *ICES CM 2018/ACOM*, pp. 859.

Figure 2a

Maps of demersal trawls stations from the Barents Sea ecosystem survey 2004-2013, showing the presence of cod by age for ages 1-10+. Each point on map is a trawl station, the red lines are the limits of the sub-regions. Due to variable survey coverage in different years, we did not include stations along the shelf break in the Northwest and northeast of the Barents Sea.

"+" on the map denotes stations without cod of that age group caught in that year

The size and color of the bubbles on the maps are proportional to the percentage of cod of that age caught at that station, out of all cod of that age caught at the survey that year.

The bar-charts are the swept area estimate of the abundance of cod from the Barents Sea ecosystem of that age group by year (2004-2013) taken from:

Johannesen E, Johnsen E, Johansen GO and Korsbrekke, K. 2019. StoX applied to cod and haddock data from the Barents Sea Ecosystem survey: Swept area abundance, length and weight at age 2004-2017. Fisken og Havet 2019-6.

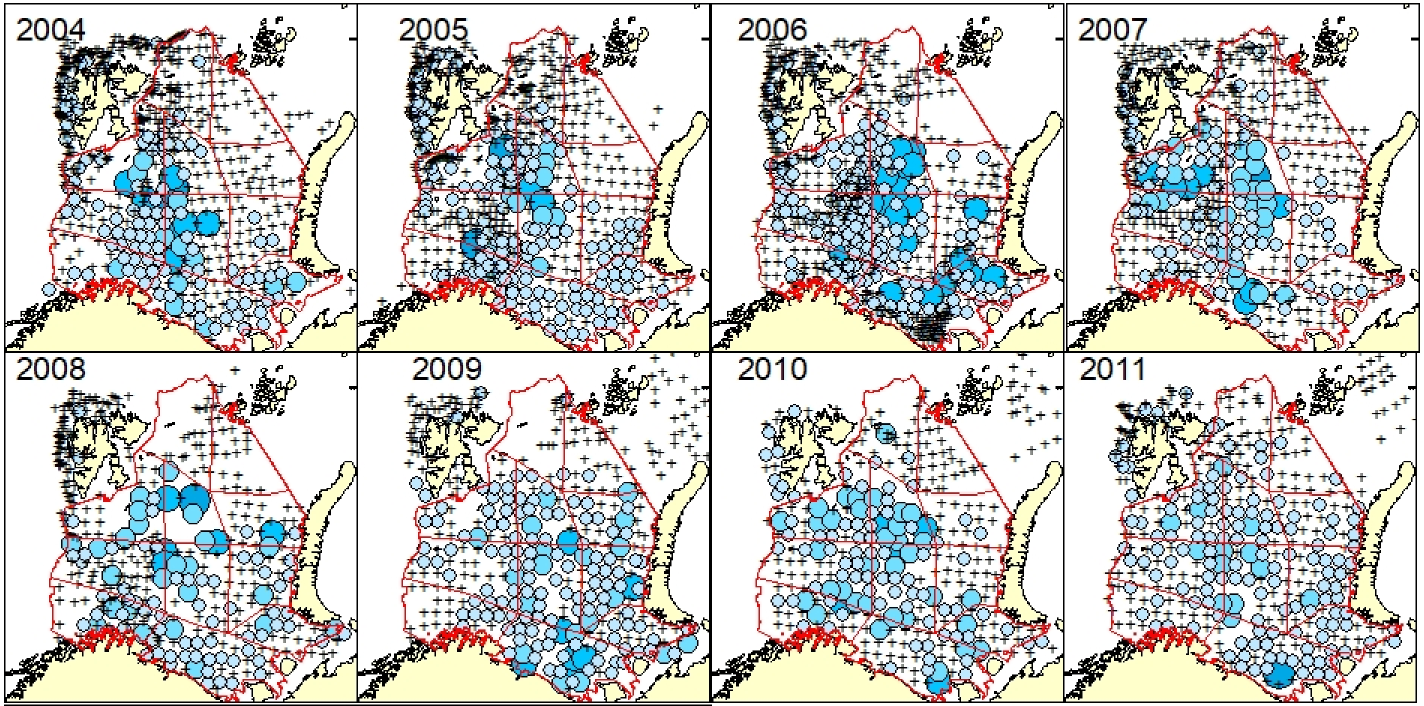
2004
2005
2006
2007
2008
2009
2010
2011
2012
2013
% of Total
0%
<1%
1-2%
2-5%
>5%

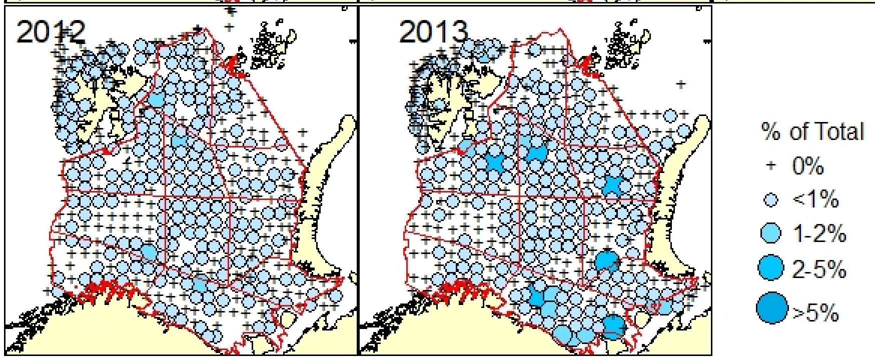

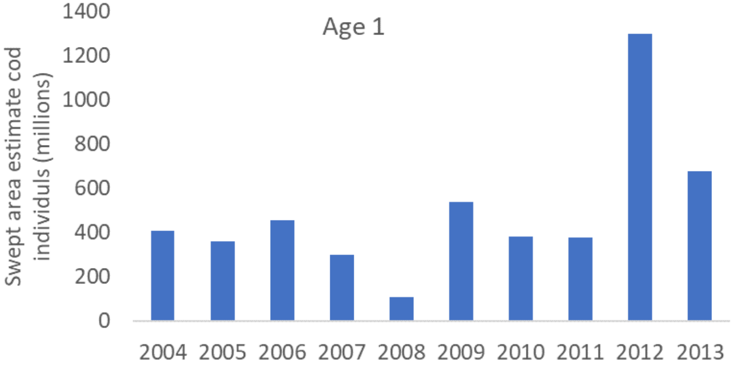
Age 1
Swept area estimate cod individuls (millions)
0
200
400
600
800
1000
1200
1400
2004
2005
2006
2007
2008
2009
2010
2011
2012
2013

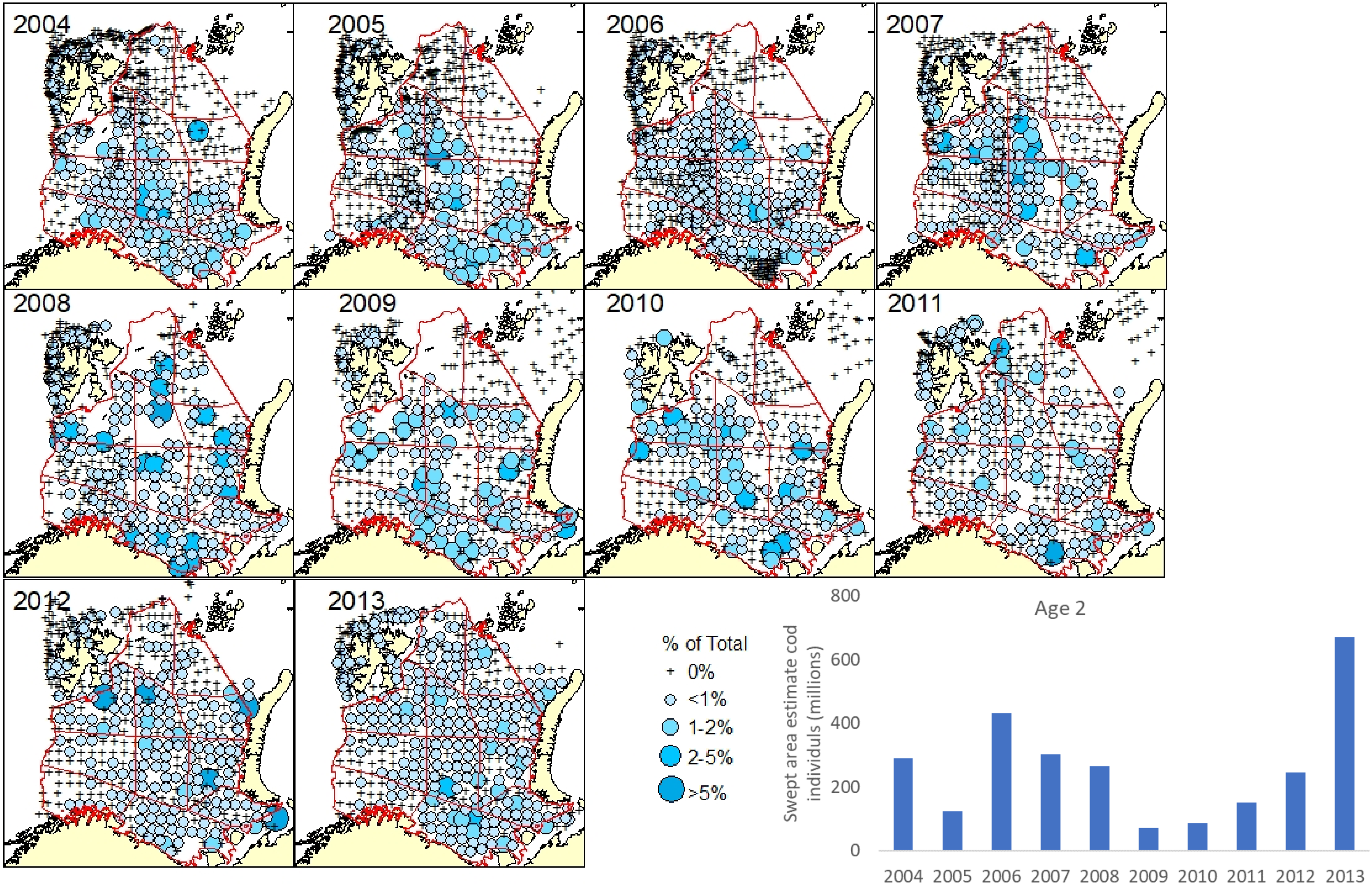
2004
2005
2006
2007
2008
2009
2010
2011
2012
2013
% of Total
0%
<1%
1-2%
2-5%
>5%
Age 2
Swept area estimate cod individuls (millions)
0
200
400
600
800
2004 2005 2006 2007 2008 2009 2010 2011 2012 2013

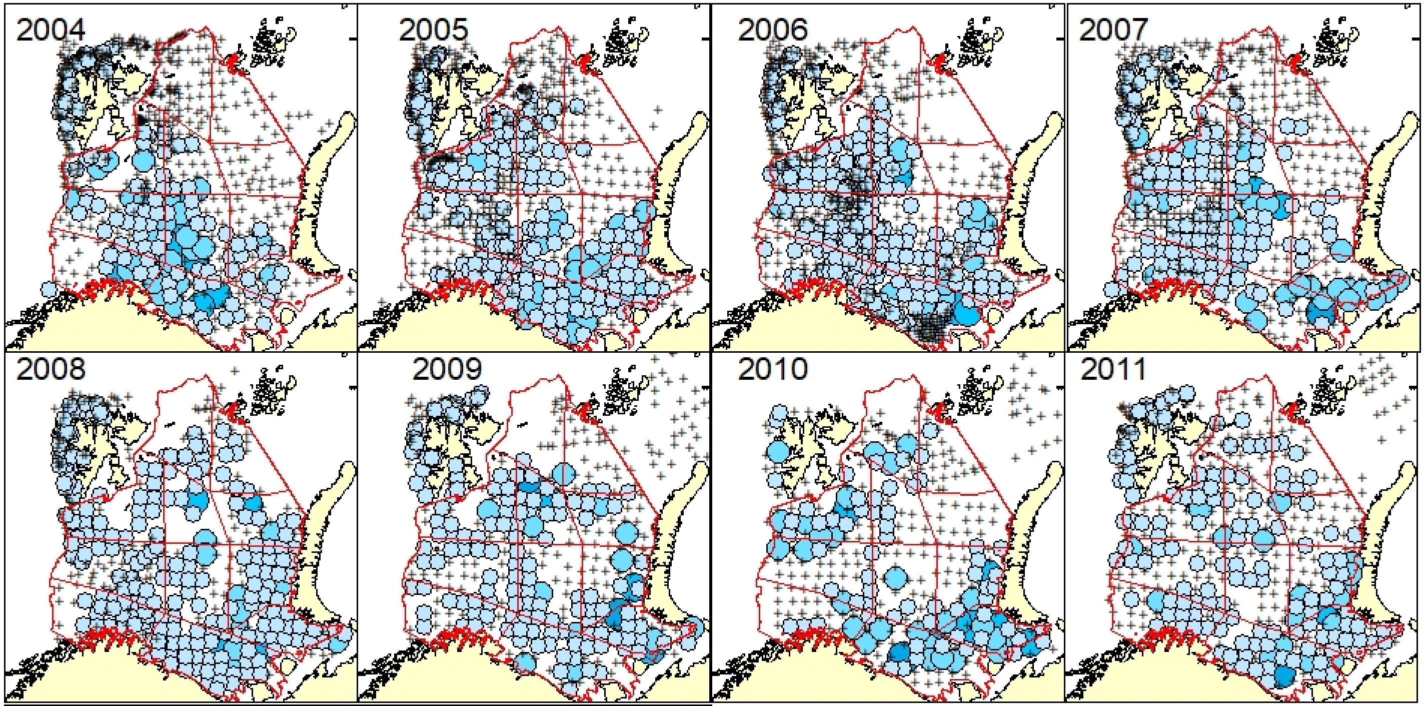
2004
2005
2006
2007
2008
2009
2010
2011

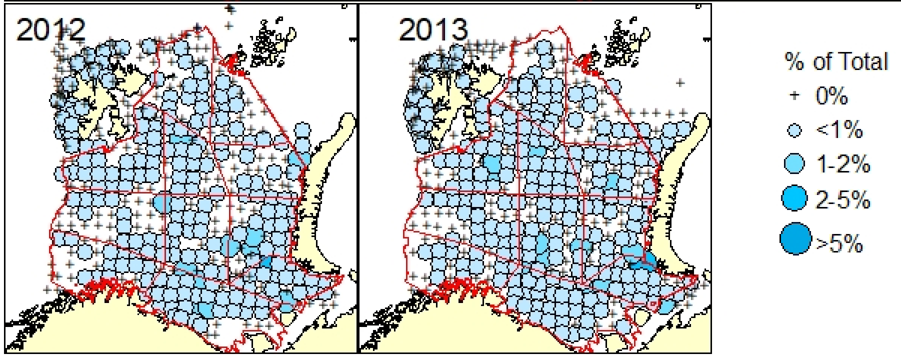
2012
2013
% of Total
0%
<1%
1-2%
2-5%
>5%

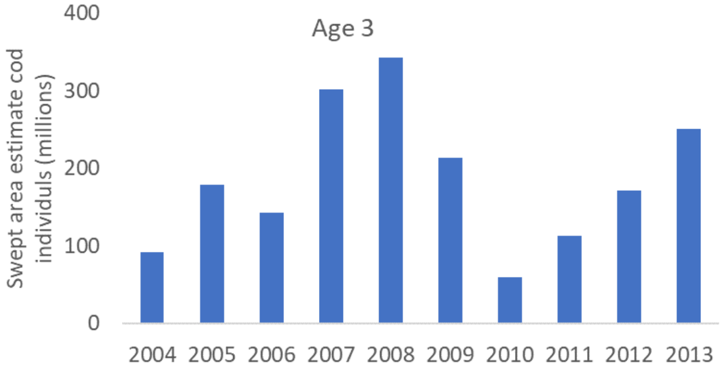
Age 3
Swept area estimate cod individuls (millions)
0
100
200
300
400
2004
2005
2006
2007
2008
2009
2010
2011
2012
2013

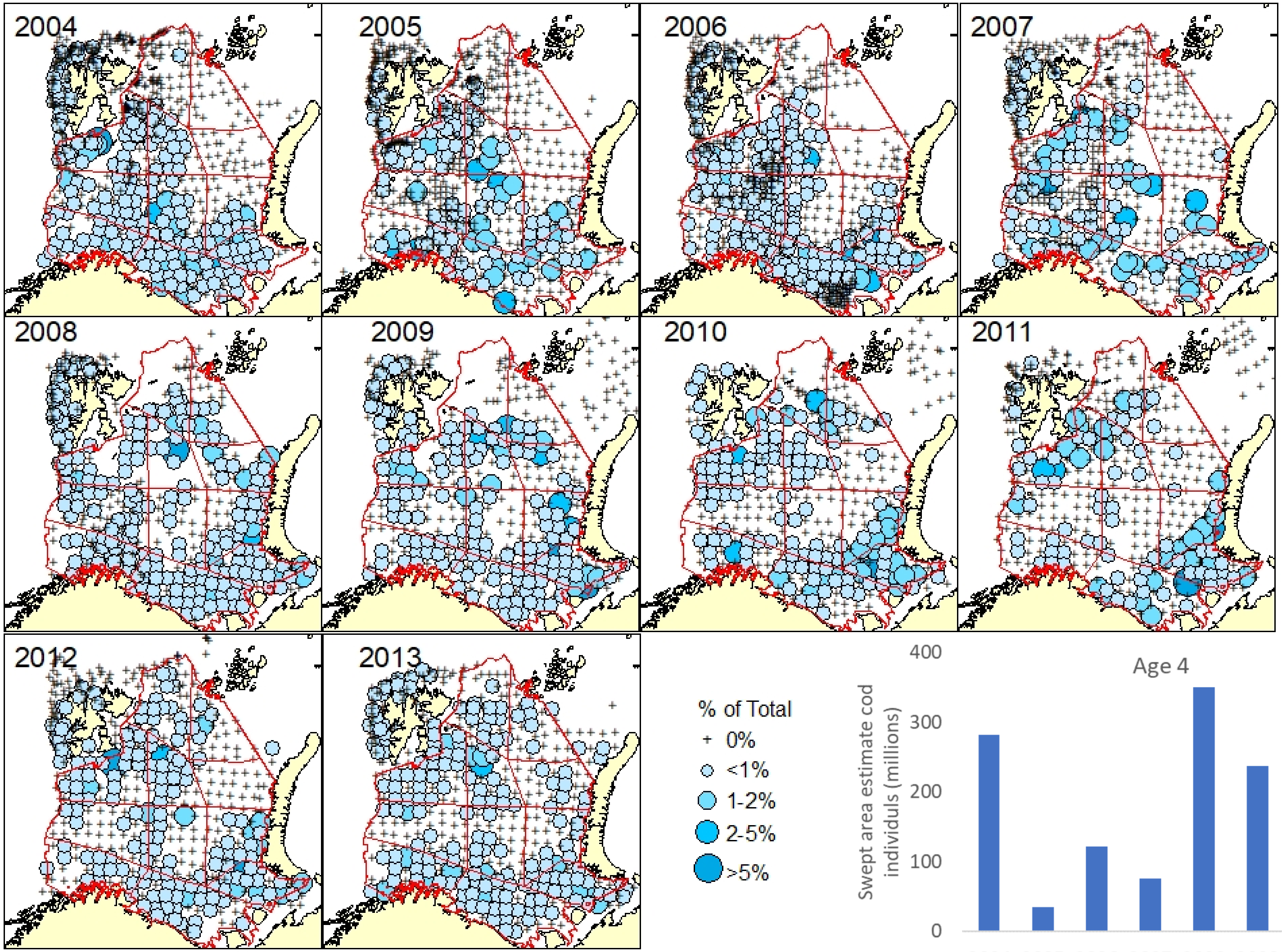
2004
2005
2006
2007
2008
2009
2010
2011
2012
2013
% of Total
0%
<1%
1-2%
2-5%
>5%

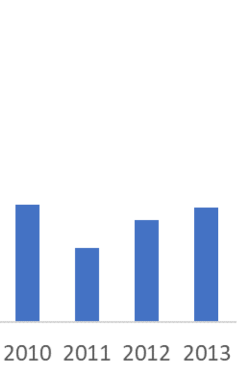
Age 4
Swept area estimate cod individuls (millions)
400
300
200
100
0
2004
2005
2006
2007
2008
2009
2010
2011
2012
2013

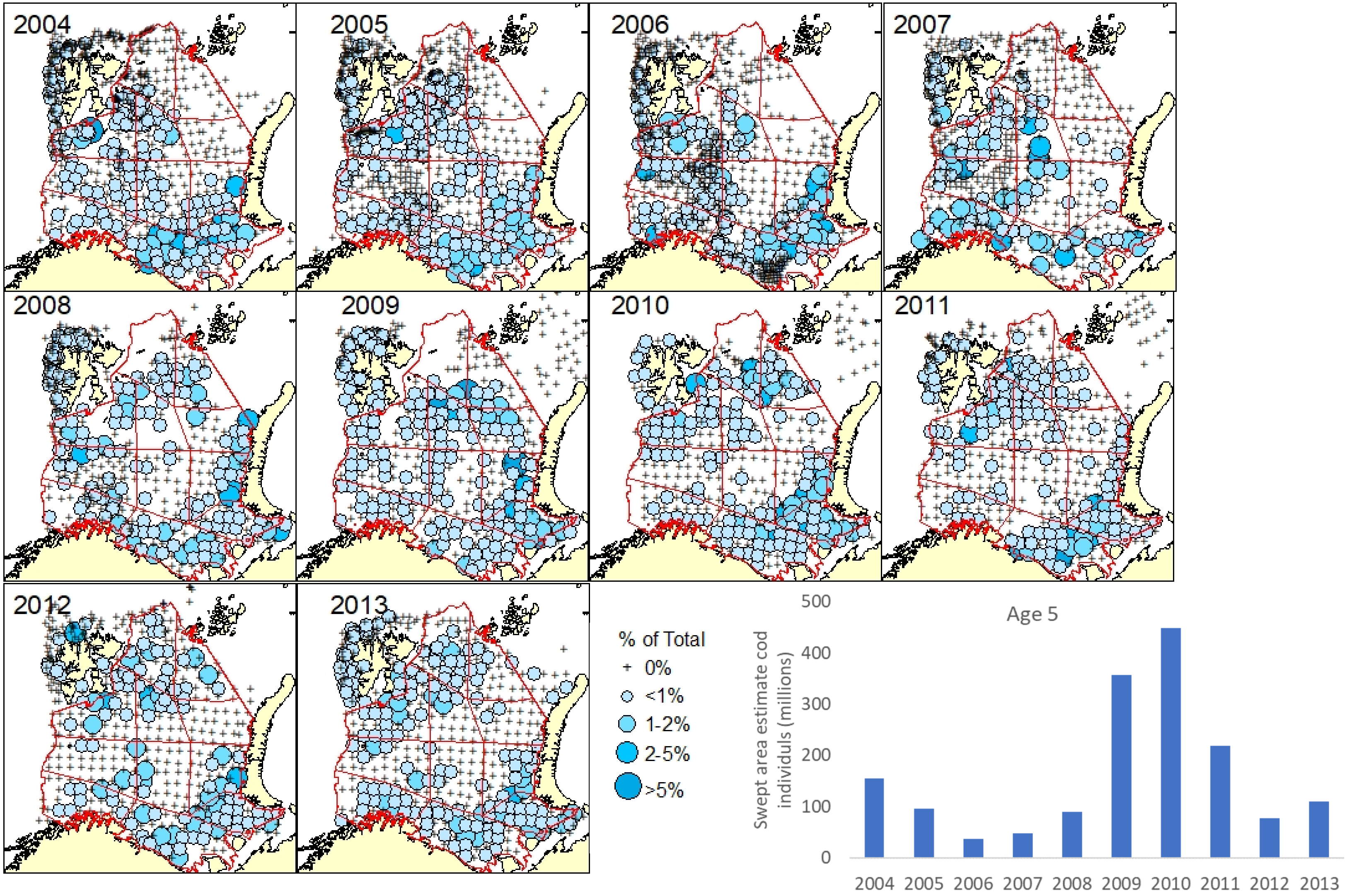
2004
2005
2006
2007
2008
2009
2010
2011
2012
2013
% of Total
0%
<1%
1-2%
2-5%
>5%
Age 5
Swept area estimate cod individuls (millions)
500
400
300
200
100
0
2004
2005
2006
2007
2008
2009
2010
2011
2012
2013

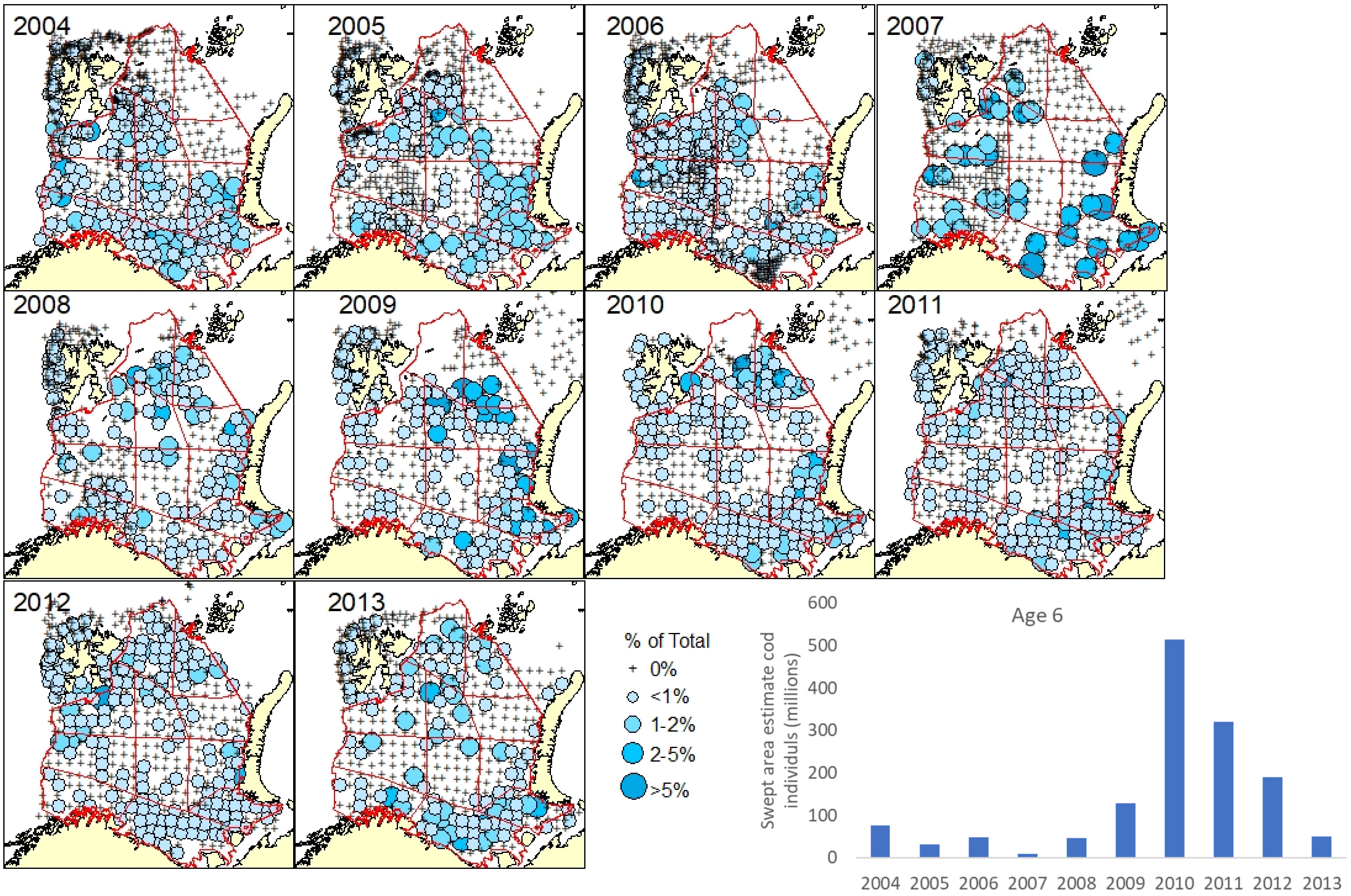
2004
2005
2006
2007
2008
2009
2010
2011
2012
2013
% of Total
0%
<1%
1-2%
2-5%
>5%
Age 6
Swept area estimate cod individuls (millions)
0
100
200
300
400
500
600
2004 2005 2006 2007 2008 2009 2010 2011 2012 2013

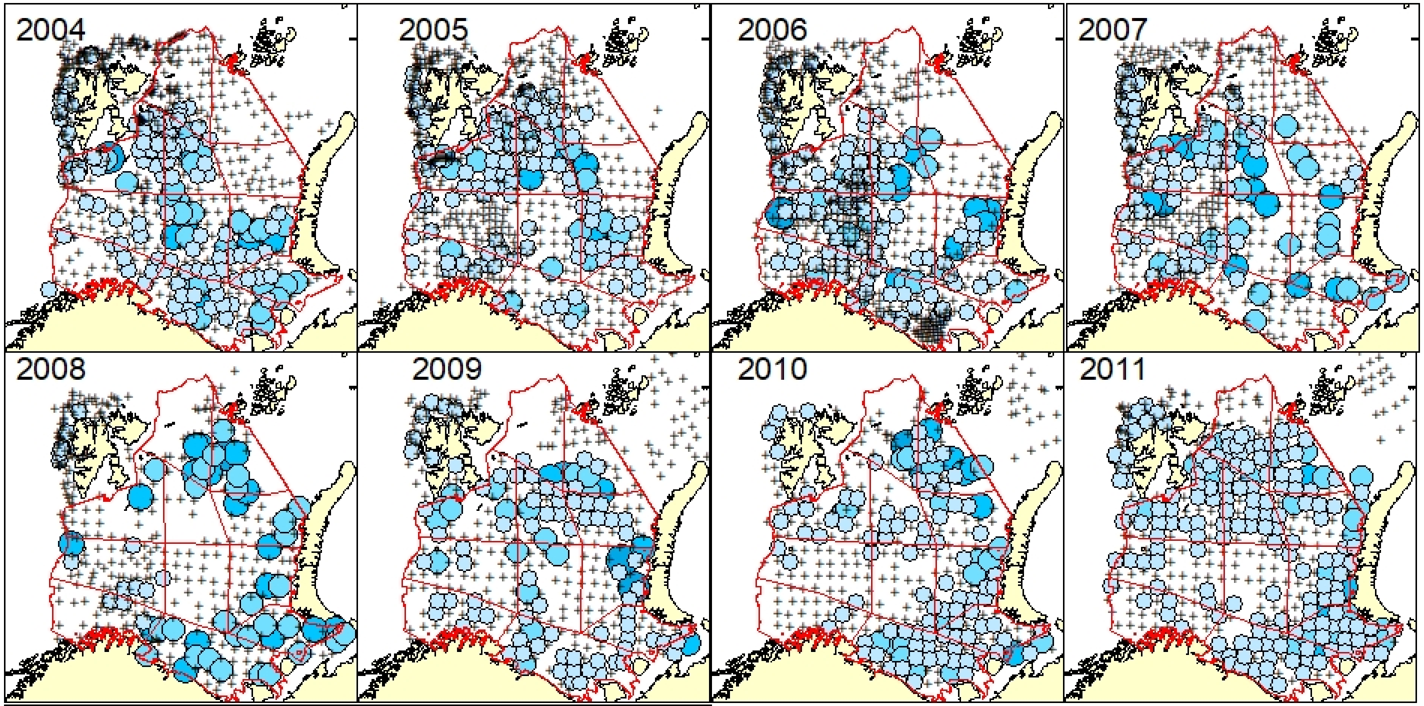
2004
2005
2006
2007
2008
2009
2010
2011

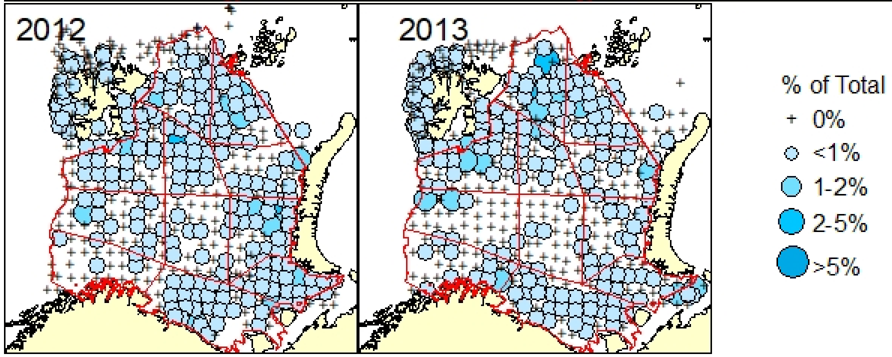
2012
2013
% of Total
0%
<1%
1-2%
2-5%
>5%

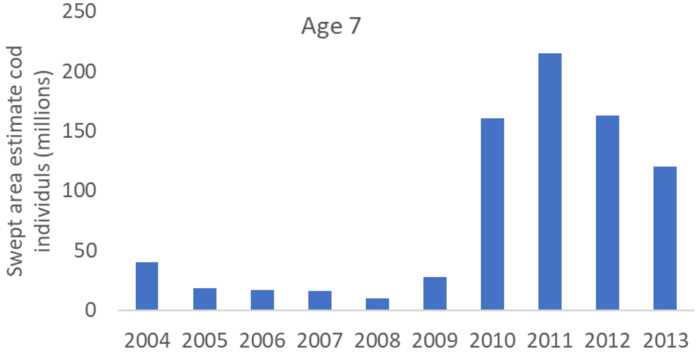
Age 7
Swept area estimate cod individuls (millions)
0
50
100
150
200
250
2004
2005
2006
2007
2008
2009
2010
2011
2012
2013

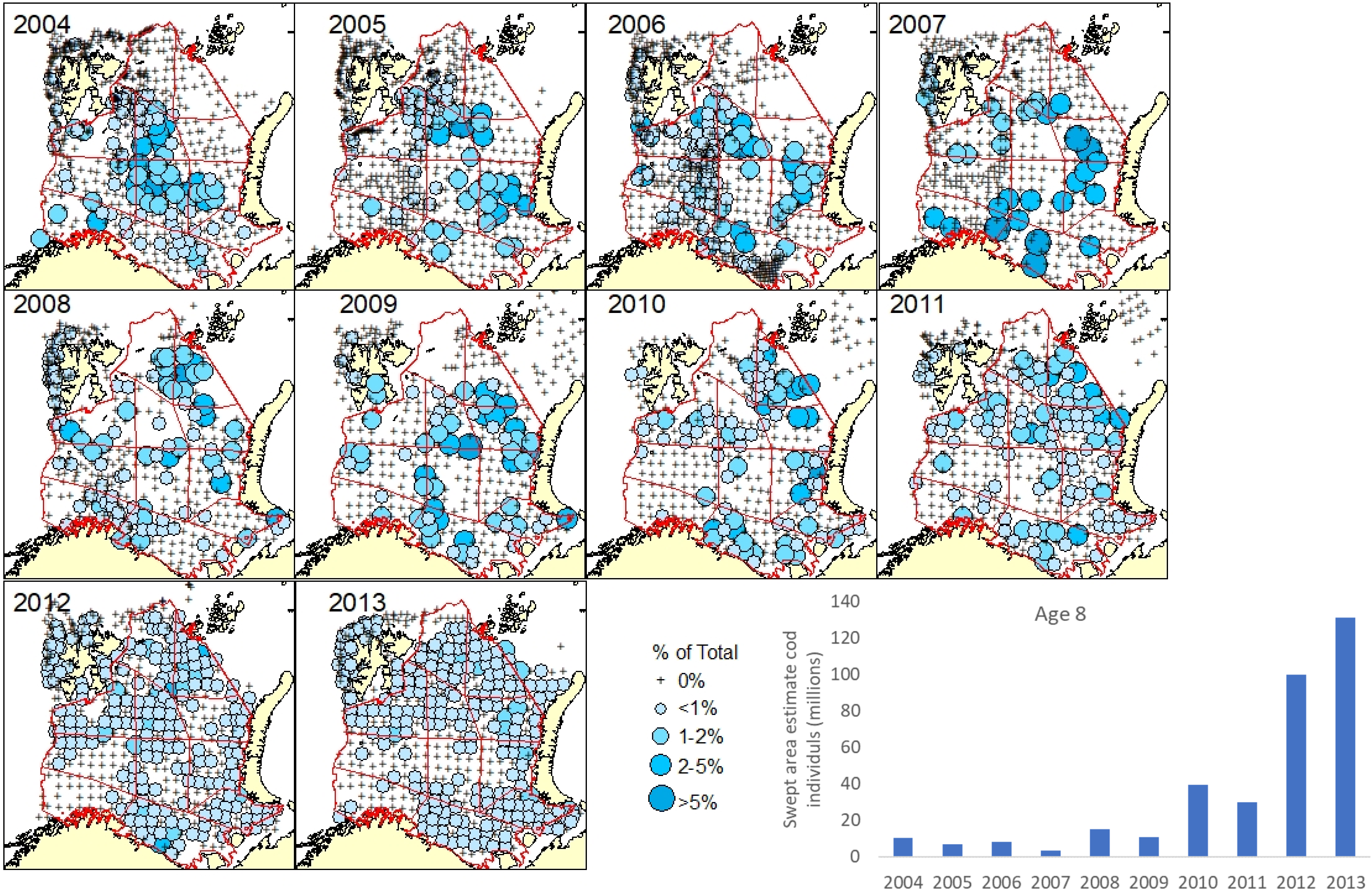
2004
2005
2006
2007
2008
2009
2010
2011
2012
2013
% of Total
+ 0%
<1%
1-2%
2-5%
>5%
Age 8
Swept area estimate cod individuls (millions)
140
120
100
80
60
40
20
0
2004
2005
2006
2007
2008
2009
2010
2011
2012
2013

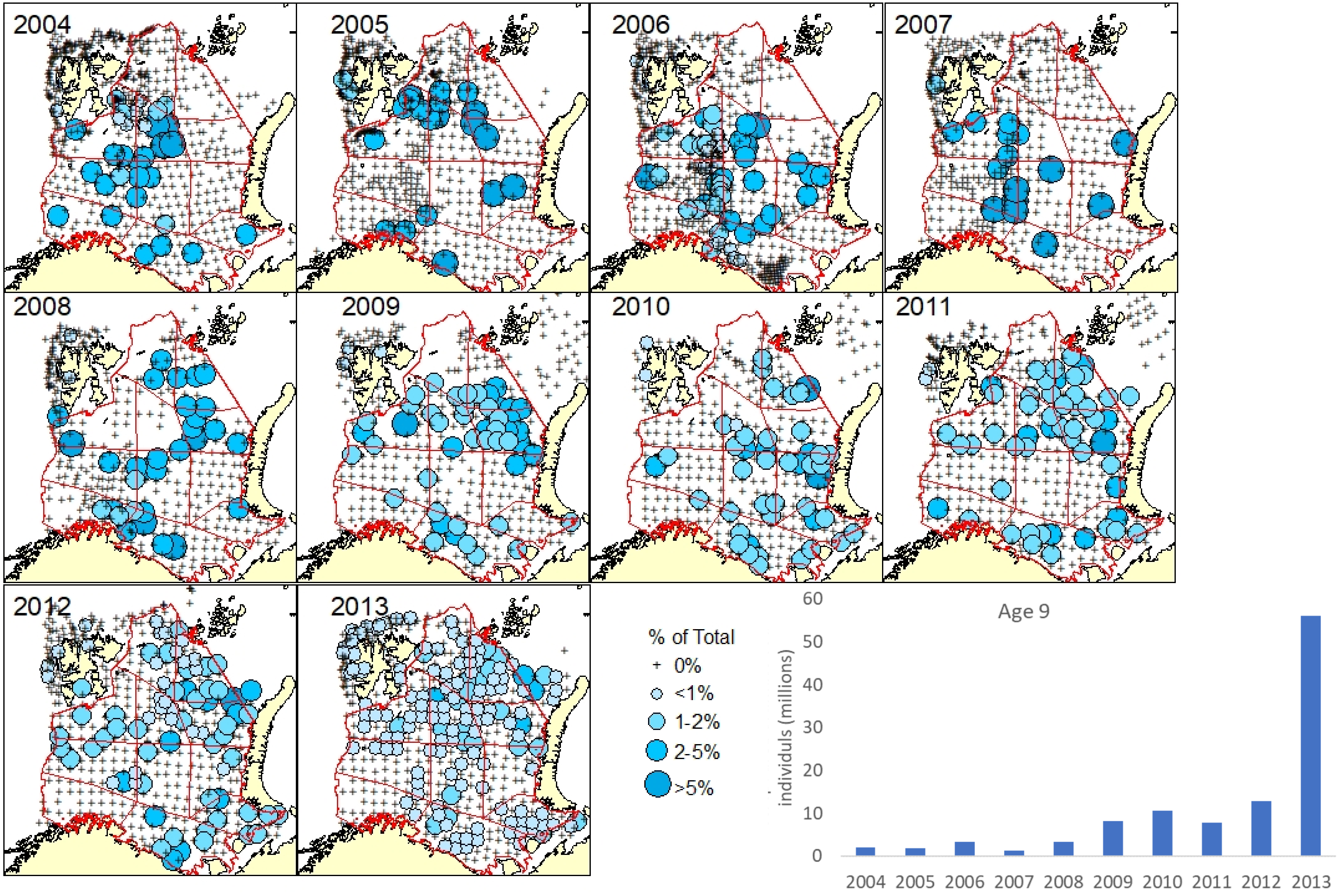
2004
2005
2006
2007
2008
2009
2010
2011
2012
2013
% of Total
+ 0%
<1%
1-2%
2-5%
>5%
Age 9
individuls (millions)
60
50
40
30
20
10
0
2004
2005
2006
2007
2008
2009
2010
2011
2012
2013

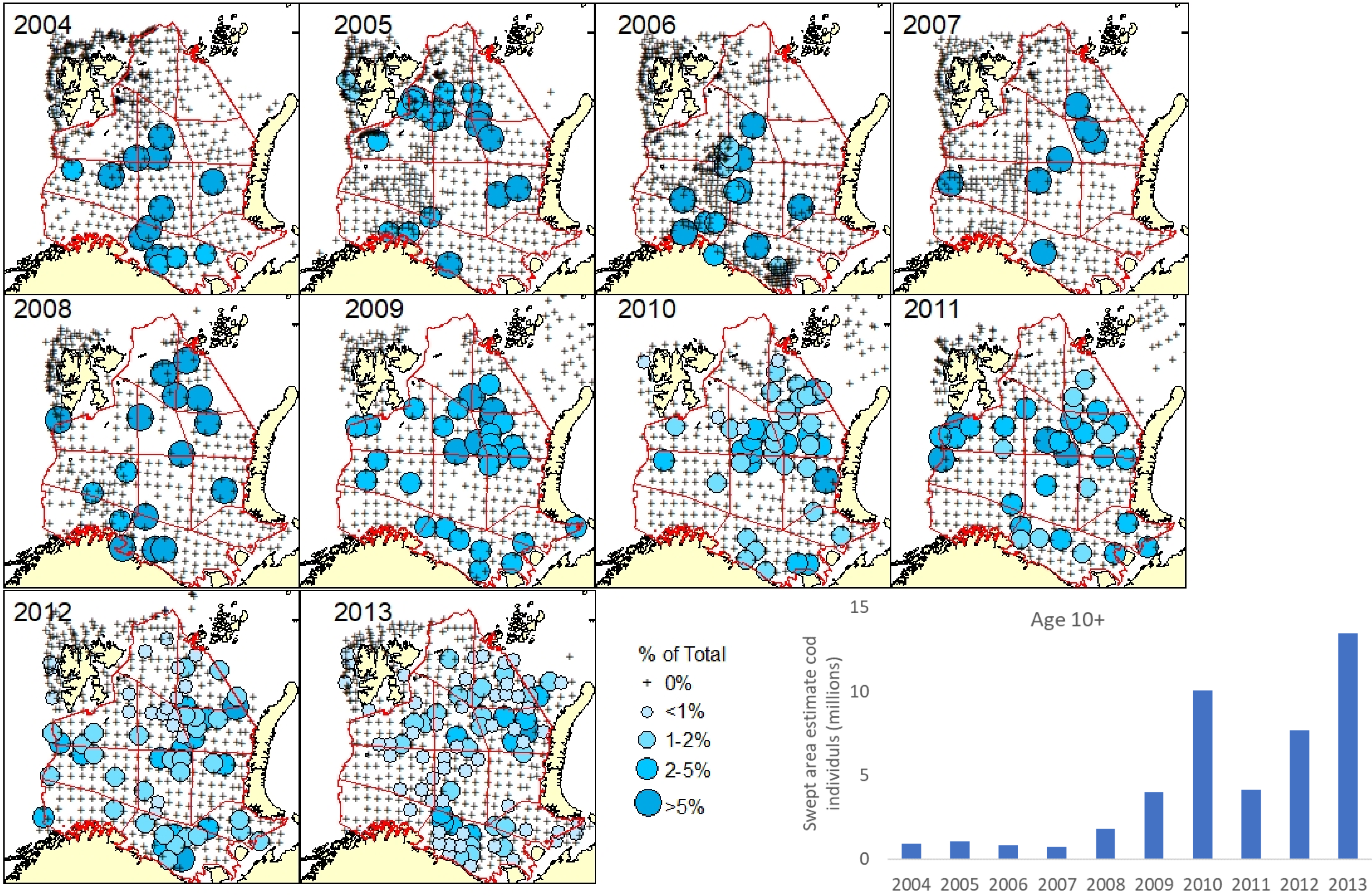
2004
2005
2006
2007
2008
2009
2010
2011
2012
2013
% of Total
0%
<1%
1-2%
2-5%
>5%
Age 10+
Swept area estimate cod individuls (millions)
0
5
10
15
2004 2005 2006 2007 2008 2009 2010 2011 2012 2013

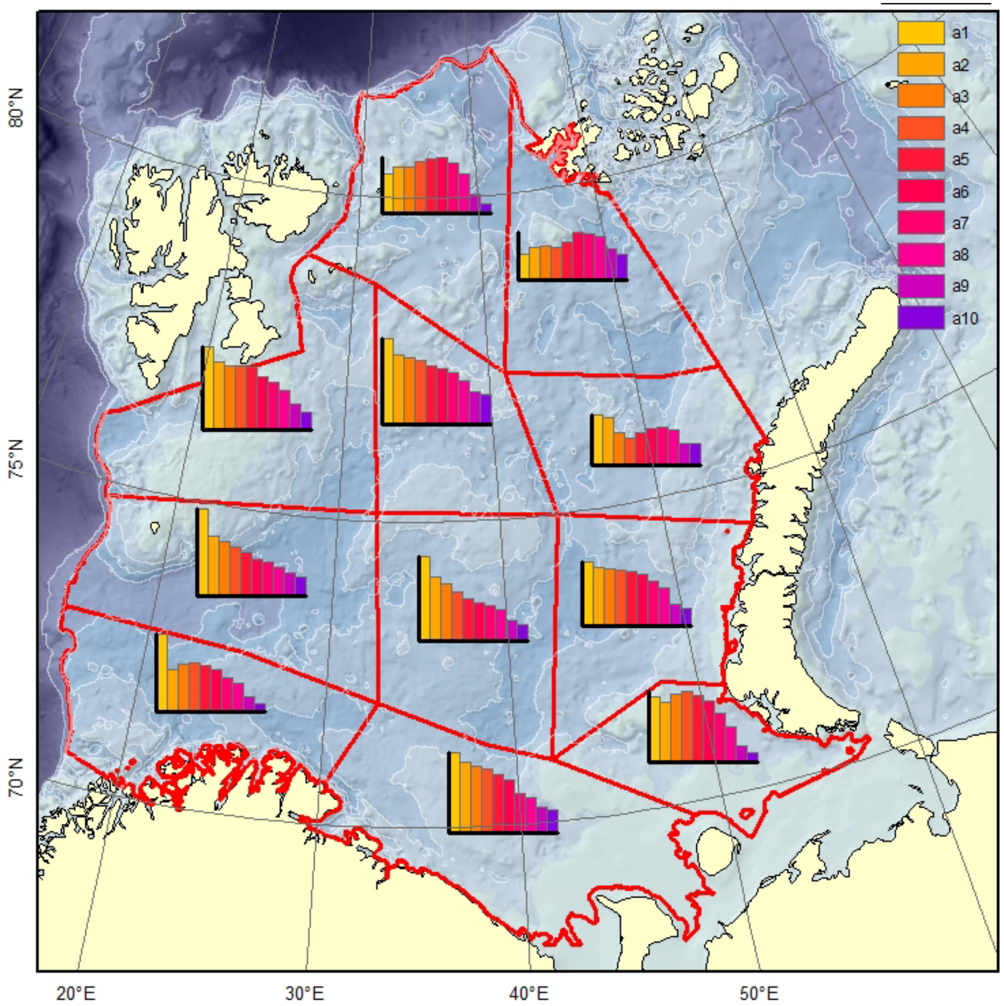

Fig. S2b

Average log cod abundance by age and sub-region

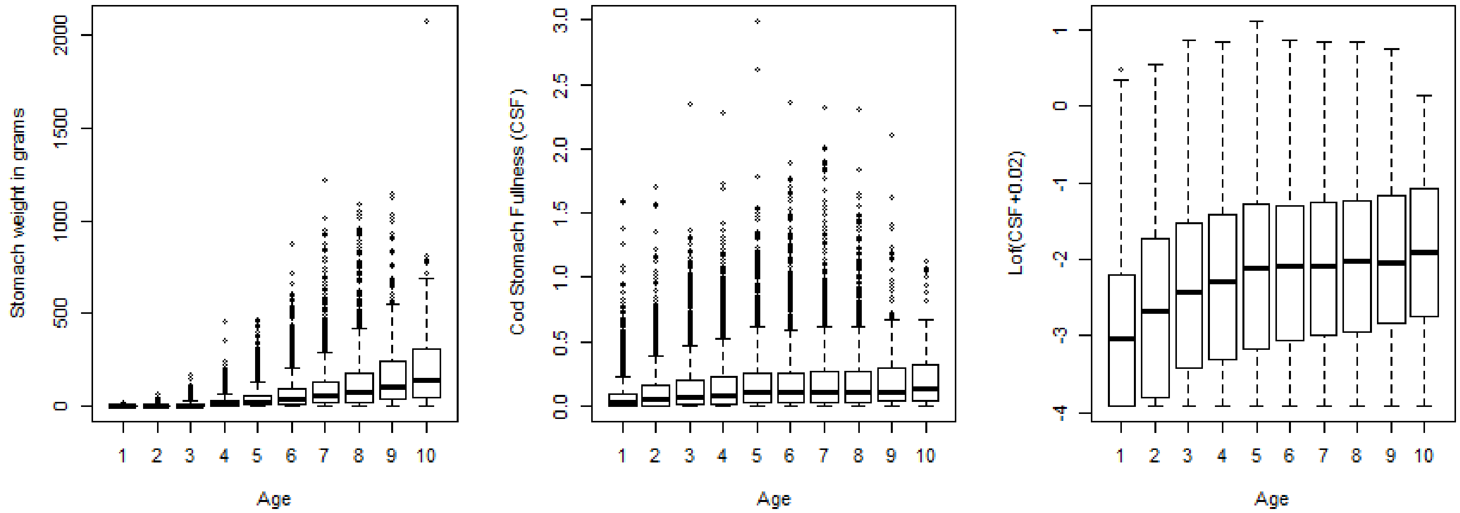

Figure S3. Stomach data by age group. Please note that the Age 10 group also includes older cod.

Left: Raw data, each point is the weight of the stomach content from one individual cod.

Middle: Cod Stomach Fullness (CSF): weight of the cod stomach multiplied by cod lenght cubed and multiplied by 10000, a common way to standardize stomach data to acount for fish size, see e.g. Dalpadado, P. & Bogstad, B. (2004) Diet of juvenile cod (age 0-2) in the Barents Sea in relation to food availability and cod growth. Polar Biology, 27, 140-154.

Right: Box Cox transformed CFS

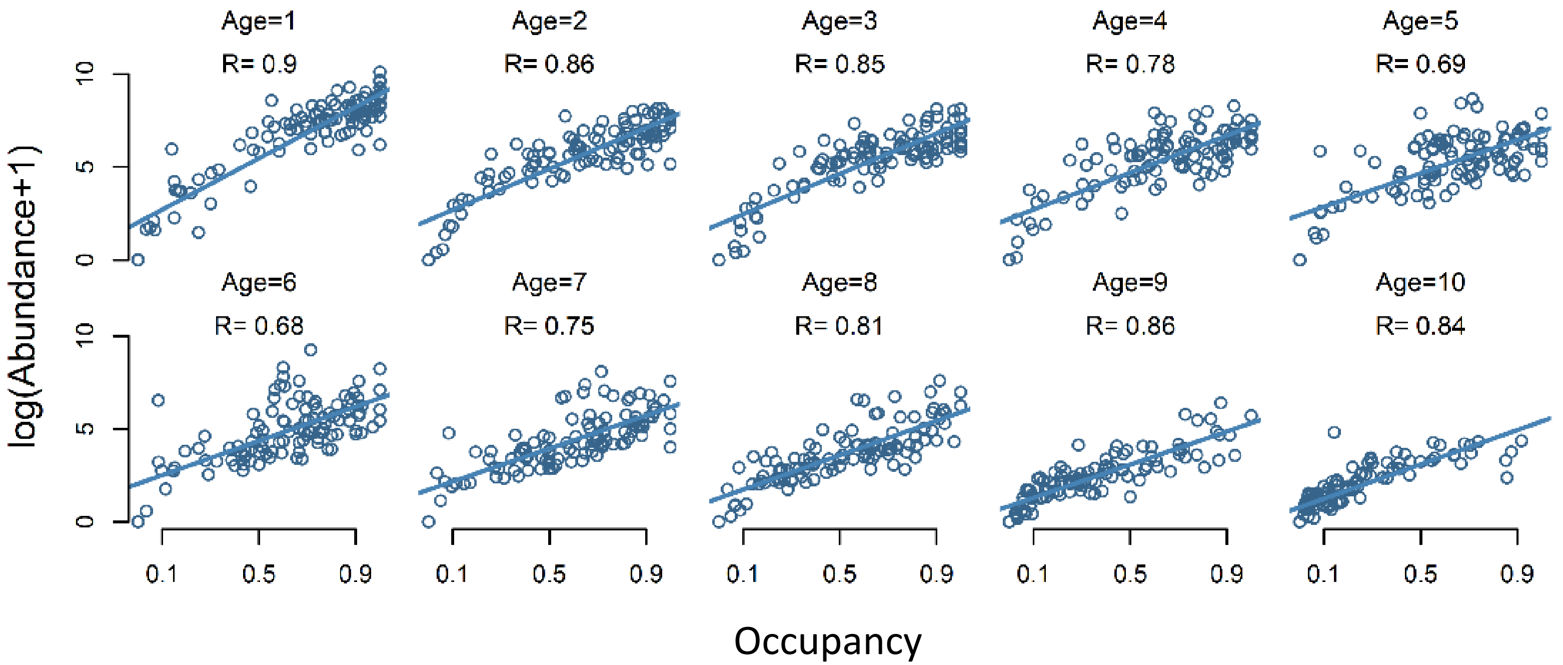

Figure S4. Cod occupancy vs abundance for each age group, age 10 include all cod 10 years or older, n=110 (10 years x 11 sub-regions).

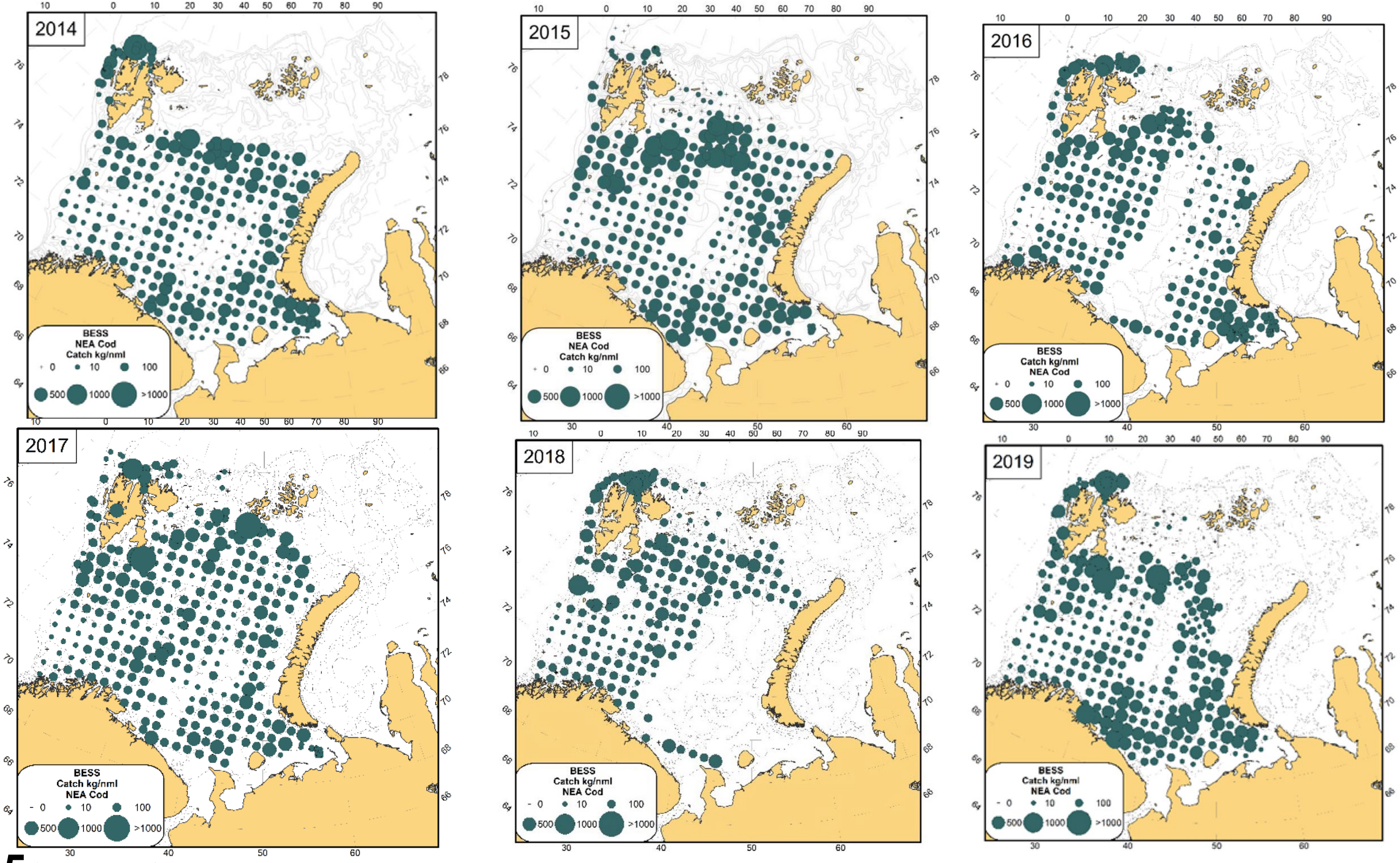

Figure S5. Maps showing the catch rate of cod by station from the Barents Sea ecosystem surveys the years after the study

Taken from:

Eriksen, E. 2015. (Ed.) Survey report from the joint Norwegian/Russian ecosystem survey in the Barents Sea and adjacent waters, August-October 2014. IMR/PINRO Joint Report Series, No. 1/2015, 153 pp. ISSN 1502-8828

Prozorkevich, D. Sunnanå K. 2016 (Eds.) Survey report from the joint Norwegian/Russian ecosystem survey in the Barents Sea and adjacent waters, August-October 2015. IMR/PINRO Joint Report Series, No. 1/2016, 77 pp. ISSN 1502-8828

Prozorkevich, D. and Sunnanå K. 2017 (Eds.) Survey report from the joint Norwegian/Russian ecosystem survey in the Barents Sea and adjacent waters, August-October 2016. IMR/PINRO Joint Report Series, No. 2/2017, 101 pp. ISSN 1502-8828

Prozorkevich, D., Johansen G.O., and van der Meeren, G.I. 2018 (Eds.) Survey report from the joint Norwegian/Russian ecosystem survey in the Barents Sea and adjacent waters, August-October 2017. IMR/PINRO Joint Report Series, No. 2/2018, 97 pp.

Prozorkevich, D., van der Meeren G.I. (Eds) 2019. Survey report from the joint Norwegian/ Russian ecosystem survey in the Barents Sea and adjacent waters August- October 2018. IMR/PINRO Joint Report Series, 2-2019, 93pp.

Prozorkevich, D., van der Meeren G.I. (Eds) 2020.Survey report from the joint Norwegian/ Russian ecosystem survey in the Barents Sea and adjacent waters August-October 2019. IMR/PINRO Joint Report Series, 1-2020, 93pp.
